# scVAEDer: integrating deep diffusion models and variational autoencoders for single-cell transcriptomics analysis

**DOI:** 10.1186/s13059-025-03519-4

**Published:** 2025-03-21

**Authors:** Mehrshad Sadria, Anita Layton

**Affiliations:** 1 Department of Applied Mathematics, University of Waterloo, Waterloo, ON Canada; 2 Cheriton School of Computer Science, University of Waterloo, Waterloo, ON Canada; 3 Department of Biology, University of Waterloo, Waterloo, ON Canada; 4 School of Pharmacy, University of Waterloo, Waterloo, ON Canada

## Abstract

Discovering a lower-dimensional embedding of single-cell data can improve downstream analysis. The embedding should encapsulate both the high-level features and low-level variations. While existing generative models attempt to learn such low-dimensional representations, they have limitations. Here, we introduce scVAEDer, a scalable deep-learning model that combines the power of variational autoencoders and deep diffusion models to learn a meaningful representation that retains both global structure and local variations. Using the learned embeddings, scVAEDer can generate novel scRNA-seq data, predict perturbation response on various cell types, identify changes in gene expression during dedifferentiation, and detect master regulators in biological processes.

**Supplementary Information:**

The online version contains supplementary material available at 10.1186/s13059-025-03519-4.

## Background

Recent advancements in single-cell sequencing technologies have presented an unparalleled opportunity to examine the characterization of cell types and states with unprecedented resolution and scale. Owing to this technology we can now generate vast amounts of data to explore intercellular interactions and precisely quantify genomic, transcriptomic, and other cellular-level information [1]. Single-cell RNA sequencing (scRNA-seq) can also be used in conjunction with other technologies such as CRISPR-based perturbation (Perturb-seq) to uncover the function of genes and regulatory elements [2]. Despite its potential, the technical complexity and high implementation costs of Perturb-seq have limited its widespread adoption [3].

Although scRNA-seq datasets have a high dimensional nature, their intrinsic dimensionality is often low, governed by cell regulatory networks [4]. Machine learning (ML), particularly deep learning (DL) models like autoencoders (AEs), have emerged as powerful tools for analyzing this data [5, 6]. These models excel at various tasks, including dimensionality reduction, clustering, data denoising, and batch effect correction [7–9]. In addition, there has been a surge of interest in using deep generative models, such as variational autoencoders (VAEs) and generative adversarial networks (GANs), for scRNA-seq analysis [10, 11]. By leveraging their ability to learn the underlying data distribution, these models have become valuable tools for generating realistic simulations of gene expression profiles, reconstructing cellular trajectories, and predicting cellular responses to different types of perturbations [12, 13]. While these generative models have demonstrated their potential, there are still issues that need to be addressed. Specifically, VAEs can experience posterior collapse [14], and GANs can struggle mode collapse [15], in which the learned mapping reduces significant portions of the input data to a single point in the output. Such problems are often caused by the disparity between the geometries of the prior and target data distributions [16]. The performance of these models is also affected by other issues such as training instability, vanishing gradients, and prior hole [17]. All these challenges can eventually lead to biased or low-quality results, ultimately hindering the application and efficiency of using generative models in single-cell technology [18, 19].

Recently, denoising diffusion models (DDMs) have gained significant attention as potent generative models. They show their exceptional performance surpassing other generative models not only in image synthesis but also in domains such as natural language processing, music, and protein structure generation [20–22]. In DDMs, the data undergoes a gradual perturbation through the diffusion process, then a deep neural network is trained to learn and remove the added noise in multiple steps. These methods can subsequently be employed to generate novel data in an iterative manner, starting from random noise without having issues associated with VAEs and GANs that were previously mentioned [23]. However, traditional DDMs necessitate a costly, iterative sampling process and lack a low-dimensional latent representation that can capture both high-level structure and fine-grained variations [24, 25]. They employ a Markov process to transform the data distribution through small perturbations, which demands a large number of diffusion steps in both the training and inference phases. As a result, employing DDMs alone for large datasets is often difficult or not feasible. Therefore, to enhance the quality and speed of these models, various attempts have been made. These include refining the forward process, implementing a more effective sampling method, integrating guidance from a classifier, or using the low dimensional latent space of autoencoders for training [24, 26–28].

In this study, we present scVAEDer, a new method for generating high-quality scRNA-seq data by combining the strengths of VAEs and DDMs (latent diffusion models). Instead of relying solely on VAE’s prior, our method incorporates both VAE and DDM priors to more precisely capture the distribution of latent encodings in the data. This allows for effective estimation of intermediate cell states and precise prediction of gene expression changes during the transition process from one cell type to another. Furthermore, by using vector arithmetic in the DDM space scVAEDer outperforms state-of-the-art (SOTA) methods in predicting single-cell perturbation responses for novel conditions, which are not included in the training process. Finally, we show that our method can successfully identify known and novel master regulatory genes involved in cellular reprogramming by computing the velocity of genes during the interpolation process. We evaluate the performance of our method by applying it to multiple datasets with diverse sizes and characteristics.

## Results

### Method outline

Generative models have shown great potential in analyzing single-cell data usually by finding a lower dimension which facilitates downstream tasks such as visualization, clustering, and data generation. However, they are not without limitations. For example, in VAEs one significant issue is the prior hole problem, where the distribution of all encodings of the training data does not perfectly form a Gaussian due to a mismatch between approximate posterior and prior distributions. This can negatively affect the quality of the generated data when interpolating or operating directly on the VAE’s latent space.

**Fig. 1:**
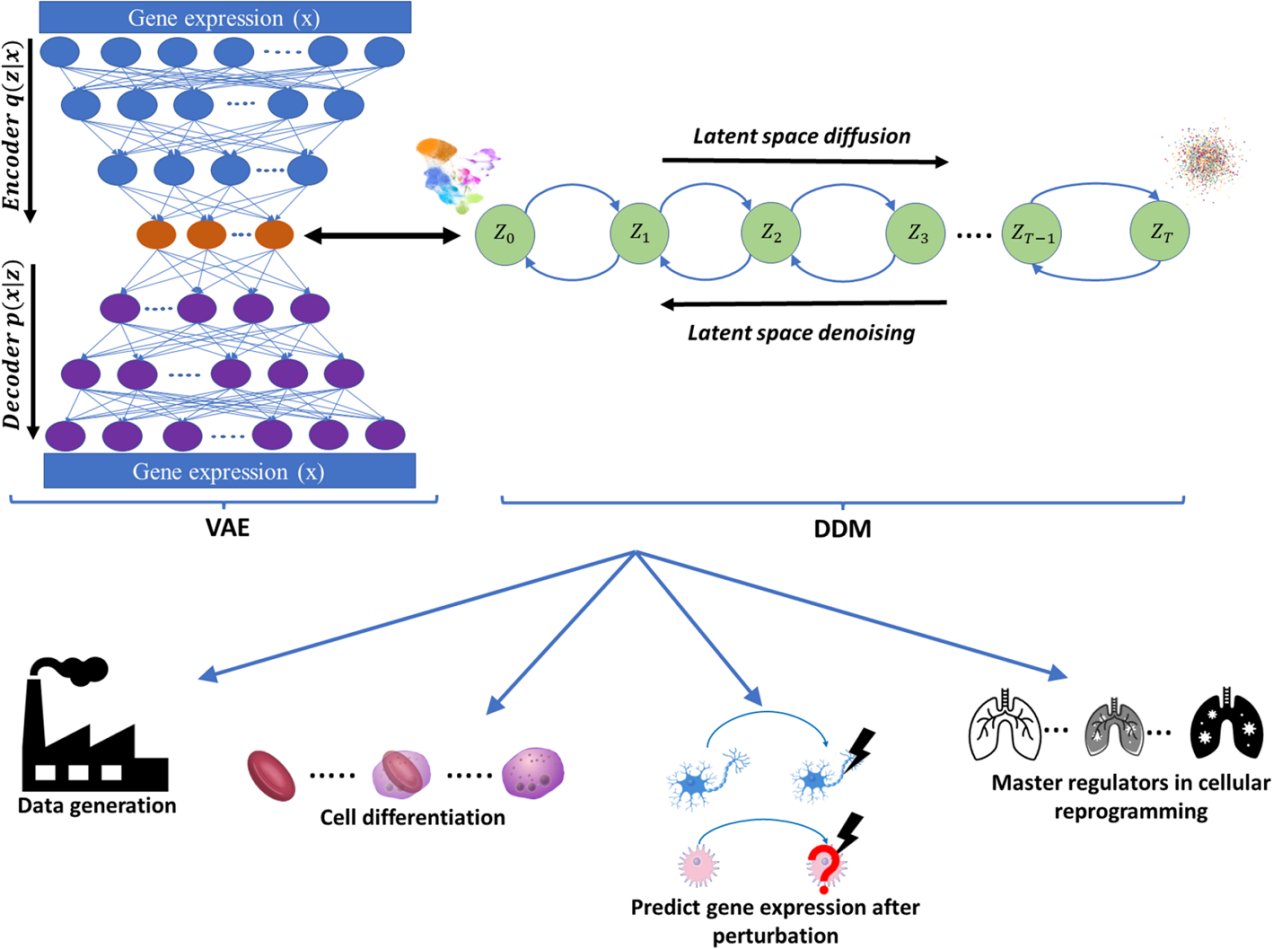
scVAEDer overview. scVAEDer integrates VAE and DDM. First, a VAE is trained using the gene expression data. Then the VAE latent embedding is used to train the DDM through the processes of latent space diffusion and denoising. Combining together the model is able to decode back the gene space with high accuracy. scVAEDer can be used for different downstream analysis tasks such as generating novel high-quality scRNA-seq data, understanding changes in gene expression during cellular differentiation, predicting the effect of perturbations on new cell types when expression data is available for multiple conditions, detecting master regulators by interpolating between different cellular states and ranking fast responder genes based on their velocity values

We develop scVAEDer that combines the strengths of VAEs and DDMs (latent diffusion models) to produce higher quality, and more reliable results without encountering VAE issues. First, it takes single-cell gene expression data as input to train a VAE. Then it uses the latent embedding generated by the VAE to train the DDM which models the data distribution by learning a denoising process iteratively (Methods). Once trained successfully, the reverse process of diffusion can be used as a generative model which maps an arbitrary Gaussian noise $N\left(0,I\right)$ to a new VAE latent representation after T successive steps (Fig. 1). Therefore, by sampling from the trained DDM and feeding it to the VAE’s decoder, realistic gene expression profiles can be generated. scVAEDer’s ability to generate high-quality data is due to its access to both VAE and DDM latent spaces, which enables capturing high-level structures and low-level stochastic variations together, consistent with leveraging latent diffusion models in other domains [24, 25, 29]. Using the trained embedding, we can also predict the effects of different perturbations and perform interpolations between cell types (see the “Methods” section for more details). We apply scVAEDer to different biological data and show that the method can accurately approximate the full distribution and trend of key genes during perturbation or cellular transition better than SOTA generative models (Fig. 1). Also, as biological systems need to respond quickly to external and internal perturbations, we believe master regulators can be discovered based on their rate of response. scVAEDer computes the velocities by looking at the changes during each interpolation step or considering the average velocity over *N* steps. Gene Set Enrichment Analysis (GSEA) on genes that are ranked based on high velocity reveals enrichment in several key pathways.

### scVAEDer generates novel realistic scRNA-seq data

**Fig. 2:**
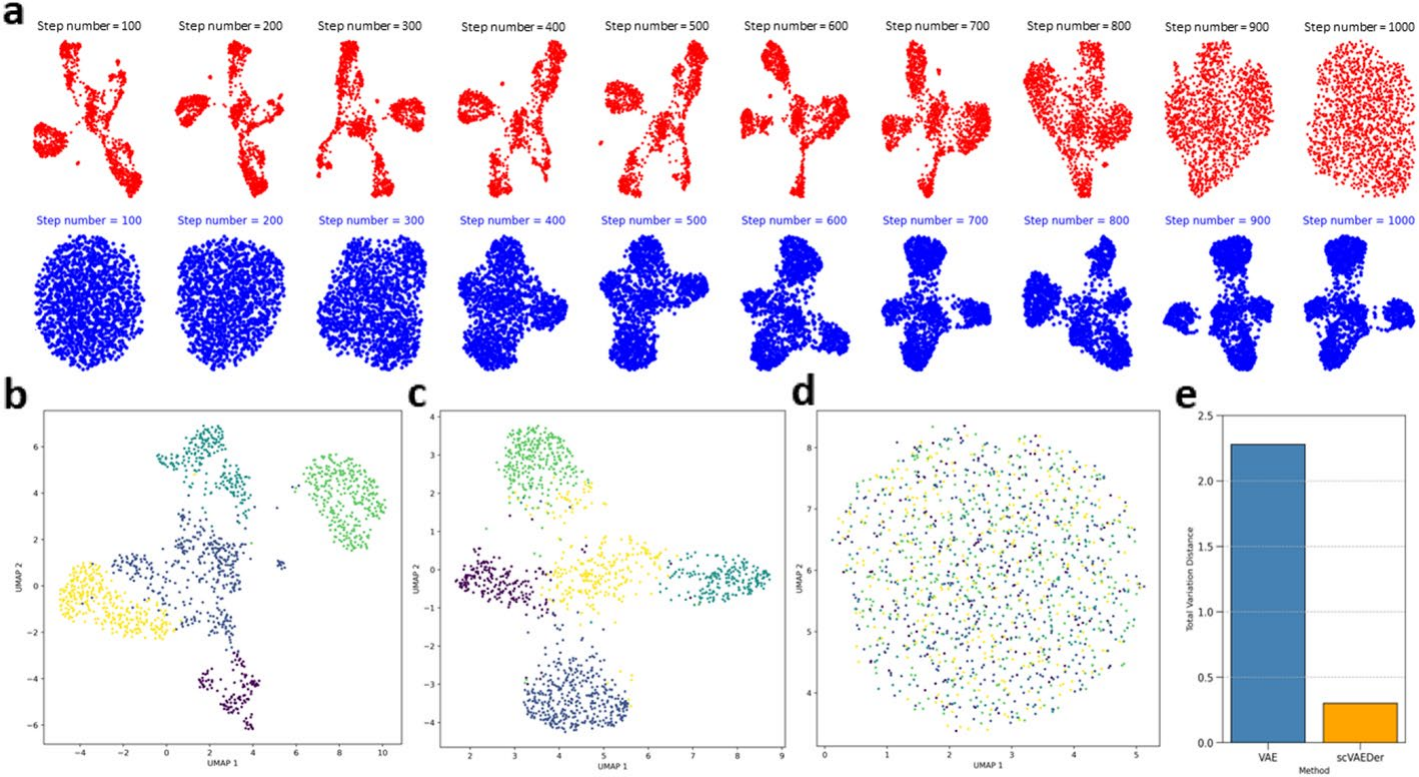
scVAEDer accurately learns the latent representation and generates new high-quality scRNA-seq data. **a** Red, forward diffusion process with 1000 steps on hematopoiesis in zebrafish as we add noise to the data; blue, reverse process as the model learns how to denoise. **b** UMAP visualization of the real data embedding. **c** Samples generated from DDM prior. **d** samples generated from the VAE. **e** Total variation distance (TVD) between latent embedding of data and samples generated from the DDM as well as VAE prior distributions

The application of DDMs in computer vision has yielded promising results for generating high-quality images. With this in mind, we investigate the potential of these models in generating single-cell gene expression data. By using scRNA-seq data of zebrafish hematopoiesis ${x}_{0}$ with 1390 cells and 1845 genes, we train a VAE and compute the latent code ${Z}_{sem}$= Encoder(${x}_{0}$). Then, ${Z}_{sem}$ is used to train a diffusion model. Figure 2a illustrates the transformation of input data into Gaussian noise through a gradual noise addition process using forward diffusion. Once DDM is trained, the reverse process can be employed to generate new samples (Fig. 2a, blue process). This involves an iterative denoising process, starting with random Gaussian noise. Increasing the number of steps in the training of DDMs results in higher-quality data generation, but also increases computational costs presenting a tradeoff between sample quality and cost (Methods for more details). To assess the performance of the data generation process, we use the fully trained diffusion model to produce new samples. Subsequently, we evaluate the quality of these generated samples. Our results show that scVAEDer generates high-quality samples with excellent fidelity to the original one, showing visual coherence and structural consistency that is important for scRNA-seq data (Fig. 2a). Furthermore, we demonstrate that the quality of data synthesis achieved by the diffusion model is superior to the ones generated by sampling from VAE’s prior. Figure 2d provides clear evidence that the structure of the embedding is lost when just using the VAE prior, while the structure of the embedding generated by the diffusion model (Fig. 2c) is similar to the real data embedding (Fig. 2b). In addition, we compute the Total Variation Distance (TVD) between the real data embedding and those generated by sampling from VAE and scVAEDer to quantitatively determine which prior can generate samples closer to the true embedding. The samples produced by scVAEDer are significantly closer to the actual values and more precisely capture the distribution of latent embedding of data in comparison to the VAE model (Fig. 2e).

### scVAEDer models the transition between cells

**Fig. 3:**
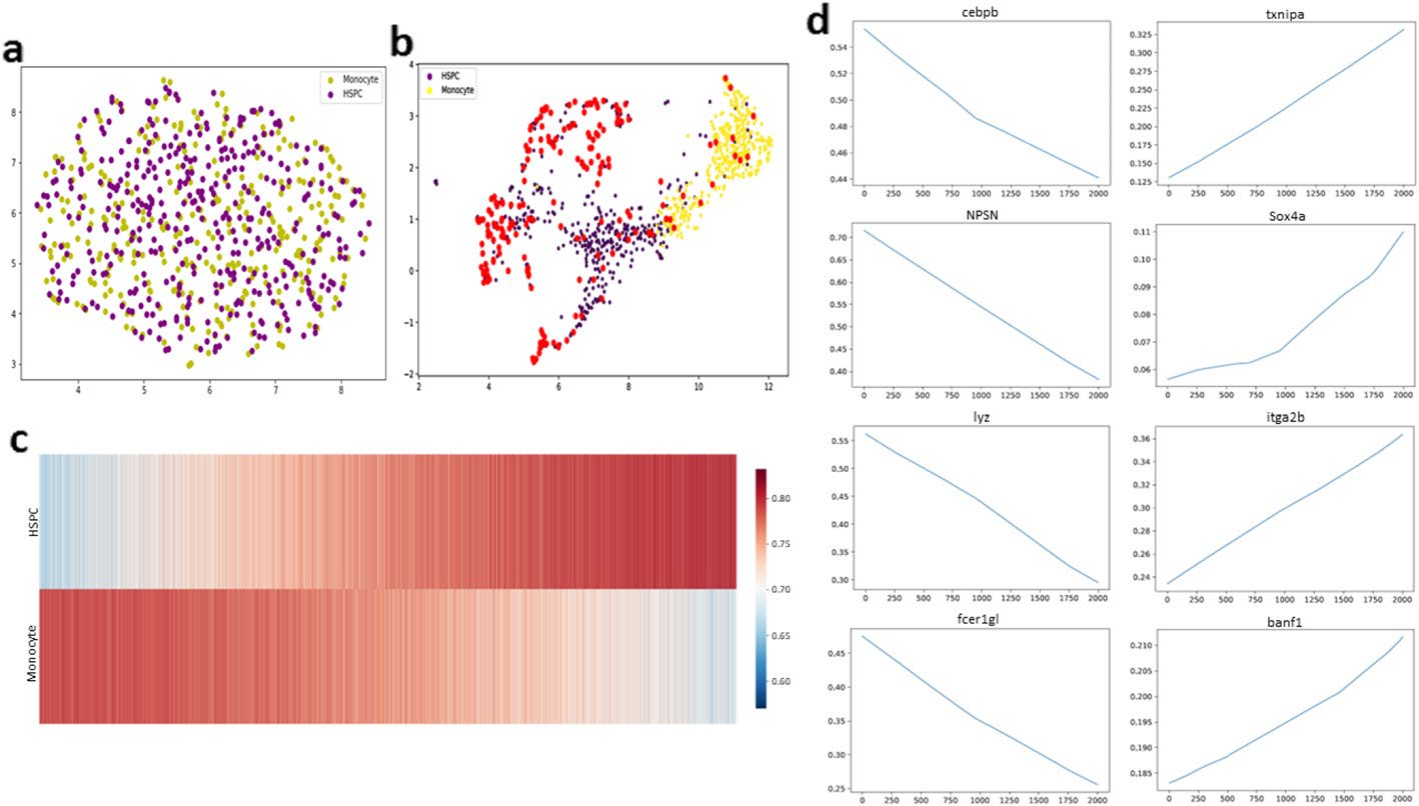
scVAEDer can be used to understand cellular dedifferentiation. **a** Mapping HSPC and monocyte cells into the latent prior of DDM. **b** Using DDM (1000 diffusion steps) and performing latent linear interpolation with 2000 equidistant samples (red dots). The absence of any sample generated in the empty region suggests that the model has learned an accurate embedding. **c** Heatmap showing the similarity between gene expression of generated states and the real average expression of HSPCs and monocytes (using 100 DDM steps before interpolation). **d** Expression of selected marker genes along the interpolation path

The ability of cells to dedifferentiate and transdifferentiate is crucial in regenerative medicine and disease treatments. Leveraging scVAEDer’s access to the entire low-dimensional embeddings, we seek to investigate gene expression changes during the cellular transition. We use hematopoiesis data, specifically focusing on the transition from monocytes to hematopoietic stem/progenitor cells (HSPC). Monocytes are a central part of the host immune system and play an essential role in immune response and inflammation functions [30]. However, the process of deriving pluripotent stem cells from monocytes is complicated and challenging [31]. Therefore, we aim to leverage scVAEDer to further investigate this process and predict how the expression of key genes changes as we interpolate between monocytes to HSPCs. To avoid the “prior hole” problem in VAEs, we interpolate in the prior space of the latent DDM (Methods). Therefore, we first compute the latent embedding for monocytes and HSPCs using the VAE encoder and use DDM forward pass to perfectly map the data to its prior (Fig. 3a). As there are no prior holes in DDM latent space, we can safely move along the interpolation path using 2000 equidistant steps between monocytes and HSPCs, and then use the reverse process to achieve the interpolation results in VAE’s latent space. These results can then be fed into the VAE’s decoder to understand changes in expression patterns as we move along the interpolation trajectory in the gene space. Interestingly, there are no samples generated in the empty region, which suggests that scVAEDer learns the correct complex dynamic of dedifferentiation (red dots in Fig. 3b).

Additionally, we compute the correlation between the cells generated by interpolation and the average gene expression of monocytes and HSPCs. During the initial interpolation stages, the cells exhibit greater similarity to monocytes; but as the process proceeds, they become more similar to HSPCs (Fig. 3c). By investigating the expression pattern of key genes during interpolation, we can gain a deeper understanding of the fundamental mechanisms driving cell dedifferentiation. As can be seen in Fig. 3d, there is a notable increase in the expression of HSPC markers in zebrafish, such as txnipa, sox4a, itga2b, and banf1 [32–35], which are critical regulators of stem cell maintenance and self-renewal. There is also a decrease in the expression of monocyte markers, such as cebpb, npsn, lyz, and fcer1gl [36–39], which are essential for the maturation and function of monocytes (Additional file 1: Fig. S1 includes the trend of more genes). This suggests that scVAEDer can capture the shifts in the activity of key genes during dedifferentiation. Moreover, by adjusting the number of diffusion steps before the interpolation process, we can modify the level of detail in the generated cells and interpolate at both fine and coarse granularities (Additional file 1: Fig. S2 and “Methods”). This finding is in agreement with the results reported by Ho et al. [21].

### scVAEDer predicts perturbation responses using feature manipulation

**Fig. 4:**
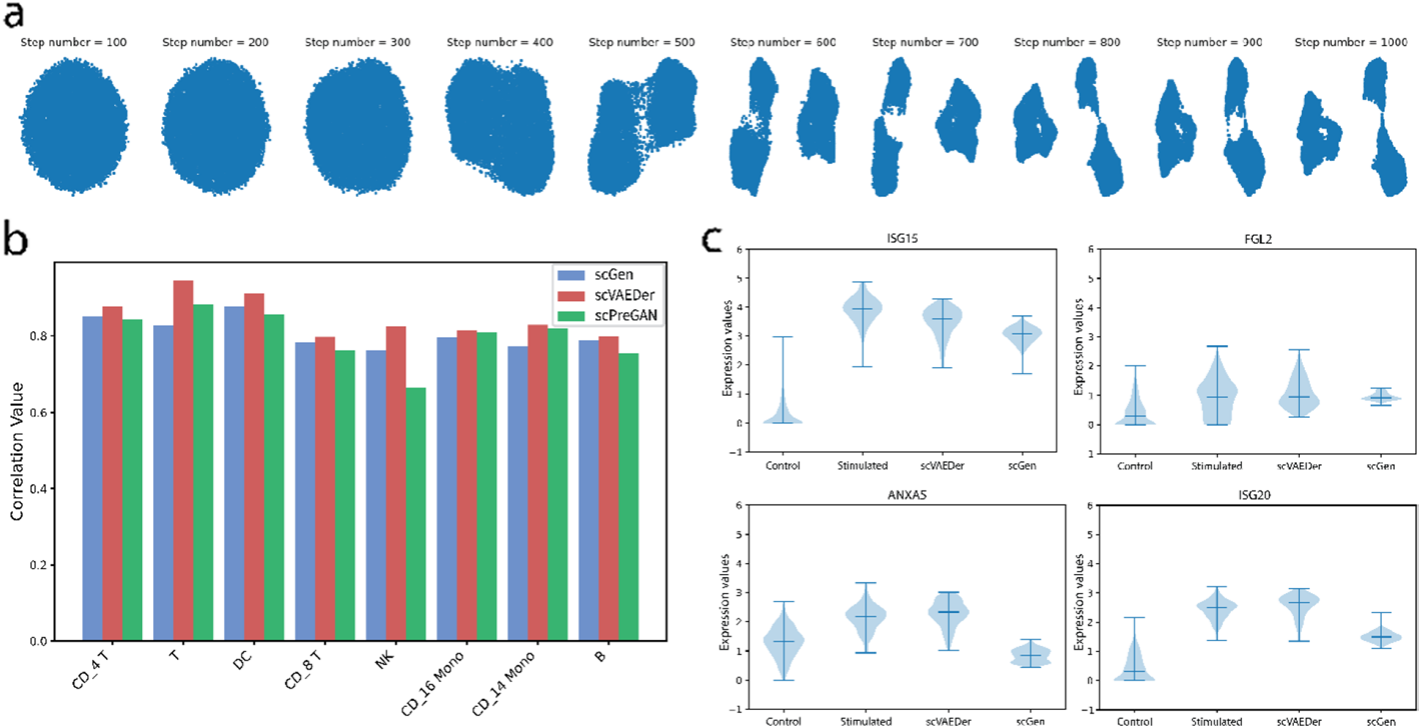
scVAEDer is more accurate than the SOTA methods in predicting perturbation responses. **a** Data generation using latent prior of DDM with 1000 diffusion steps. **b** Comparison of the correlation values of average gene expression between real and predicted cells of various cell types obtained using scGen, scPreGAN, and scVAEDer. **c** Violin plots for selected key genes across control, real stimulated, and stimulation predicted by scVAEDer and scGen in dendritic cells (DC)

By studying cell responses to specific perturbations, we can understand the underlying mechanisms of a biological process, such as gene regulation and signal transduction, as well as the mechanisms of diseases. Different types of autoencoders have been used in the exploration of the effect of various types of perturbations on cells through vector arithmetic [5, 11]. scVAEDer latent can be used to predict the refined perturbed gene expression while avoiding VAE issues. To evaluate the performance of our model in predicting perturbation response, scRNA-seq data of human peripheral blood mononuclear cells (PBMCs) stimulated with interferon (IFN-β) [40] is used for several training of scVAEDer (Fig. 4a). This dataset includes 6359 control and 7217 stimulated cells, with dramatic changes in the transcriptional profiles of immune cells induced by IFN-β stimulation. Following this initial assessment, we examine scVAEDer’s performance in predicting perturbation responses across diverse cell types. This evaluation involves comparing scVAEDer against the state-of-the-art methods scGen [11] (based on VAE) and scPreGAN [12] (based on GAN) by calculating the correlation between the predicted and real average gene expression levels for various cell types (Fig. 4b, “Methods”). Notably, scVAEDer exhibits superior performance in predicting perturbation responses compared to the other models. To further investigate scVAEDer’s performance, we conducted an extensive search to identify genes that are shown to be related and important in PBMC from patients with lupus pathogenesis. This includes genes such as ISG15, ISG20, FGL2, and ANXA5 [41–44]. We then use these genes to assess scVAEDer’s accuracy in predicting their expression distribution following perturbation for Dendritic cells (DC). Here, scVAEDer surpasses scGen in predicting the total distribution of these crucial genes, highlighting its high precision (Fig. 4c). Additional results for more key genes are provided in Additional file 1: Fig. S3a.

Furthermore, to assess the overall robustness of scVAEDer, we incorporate an additional dataset of intestinal epithelial cells after Salmonella infections and re-evaluate scVAEDer’s performance against scGen and scPreGAN (Additional file 1: Fig. S3b). This broader evaluation confirms that scVAEDer consistently outperforms other methods across various cell types.

### Disease progression and master regulators

**Fig. 5:**
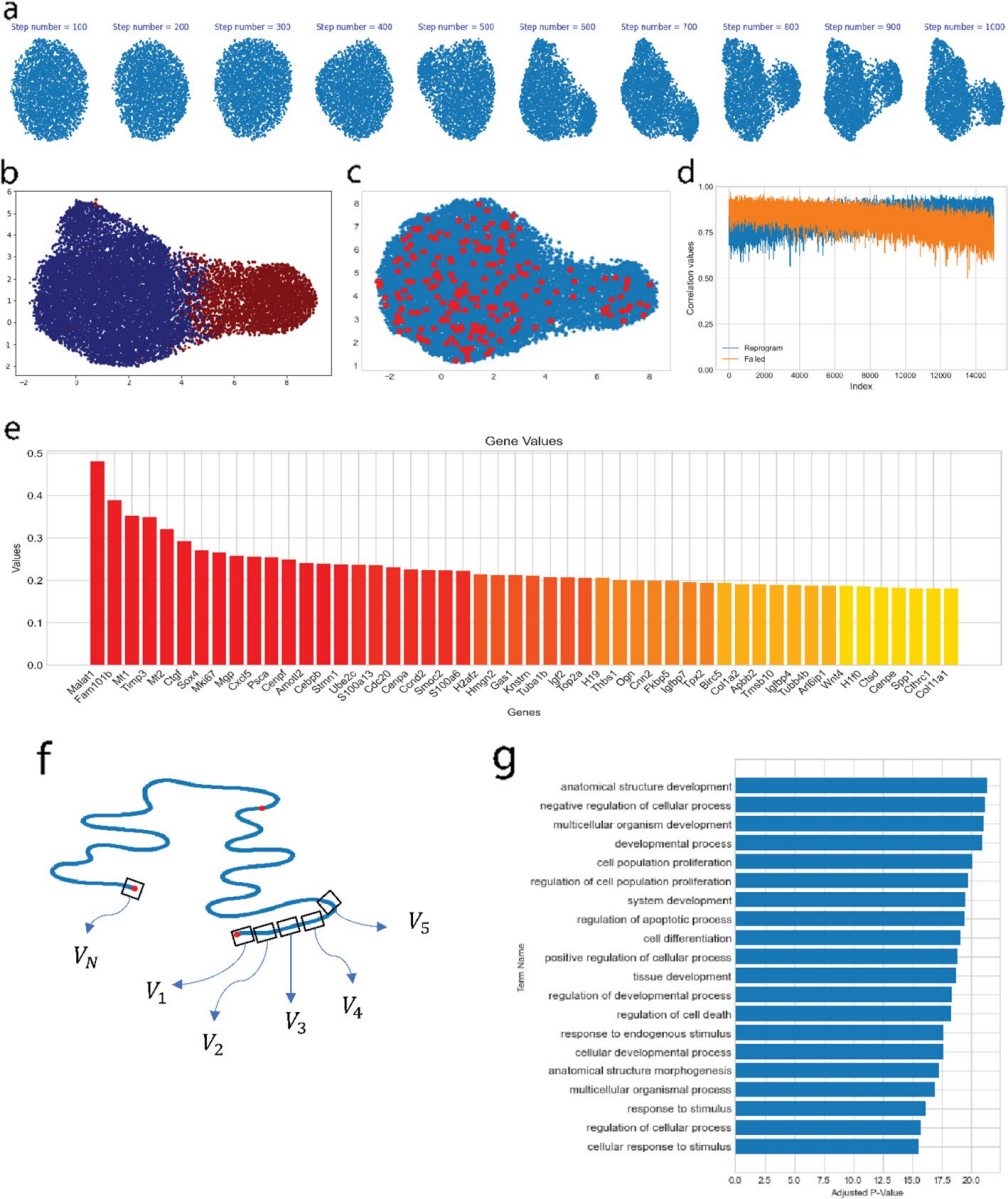
scVAEDer accurately detects master regulators during cellular reprogramming. **a** The reverse process of DDM allows the generation of high-quality reprogramming samples from random Gaussian noise (the quality of generated samples is critical for downstream analysis). **b** UMAP visualization of the data latent embedding, colored based on their state (red: failed, blue: reprogrammed). **c** Data generated by interpolating between reprogrammed and failed states, represented by red dots. Remarkably, none of the interpolated samples are found outside the real representation of data. **d** Correlation values between the new interpolated samples and the average gene expression of reprogrammed and failed cells, which demonstrates the shift in gene expression between failed and reprogrammed states. **e** Ranking of genes based on high expression differences between *t* = 0 and *t* = 3000. **f** Illustration showing the process of computing gene velocities along the interpolation path to detect master regulators. **g** Gene set enrichment analysis using 400 genes with the highest velocities (fast responders), which reveals pathways that are crucial during the cell reprogramming process

The study of specific genes known as “master regulators” is of paramount importance in the control of cell fate and differentiation, as they can be targeted to reprogram cells into desired cell types and states. As such, their detection is crucial for gaining insights into disease mechanisms and advancing the development of potential therapies. Here we investigate the changes in gene expression during the transition from failed to reprogrammed cell states and to identify master regulators involved in this process. To achieve this, we employ an approach that involves interpolating between failed and reprogrammed cell states using scVAEDer. Reprogramming data which has 18,803 cells and 28,001 genes is used to train scVAEDer after the preprocessing steps. We first validate that scVAEDer is able to accurately generate high-quality data from Gaussian noise (Fig. 5a). Then using the trained model, we obtain the latent embeddings of reprogrammed and failed cells. The UMAP visualization of the cell states in the VAE space can be seen in Fig. 5b. We use the average gene expression of failed and reprogrammed cells and interpolate in scVAEDer’s diffusion prior. Then the reverse process maps the interpolation results back into VAE’s space (Fig. 5c). The correlation values are computed between each interpolation step and the average gene expression of reprogrammed and failed cells. As we move along the interpolation path, the gene expression becomes more similar to the reprogrammed state and less like the failed state (Fig. 5d). We are also able to control the level of granularity during the interpolation process by adjusting the number of steps of DDM before interpolation (Methods). Additionally, to understand marker genes during this transitional process, we calculate the absolute difference between the gene expression at time *t* = 0 and *t* = 3000 (corresponds to 20% of the total 15,000 as it corresponds to the global structural changes in the data). The genes with the highest absolute scores are displayed in Fig. 5e. This allows us to identify both known and previously unknown gene markers linked to the reprogramming of fibroblasts [45–48].

Next, we use scVAEDer to detect the master regulators involved in this process, by computing the gene velocities, given by the rate of changes in gene expression during interpolation (Fig. 5f). We believe gene velocities are informative as they allow us to identify crucial genes that should undergo rapid changes in order to play their central role in a specific biological process. By ranking genes based on their absolute velocity values, we detect key genes in this process, including Wnt4, Ptma, and FoxA1, which have been shown to be important in reprogramming and regeneration processes (list of the top 15 genes and their corresponding functions [49–58] can be found in Additional file 1: Table S1). As a biological system needs different genes and pathways to be activated in order to succeed in different parts of the biological process, our approach makes it possible to detect master regulators in each step. Furthermore, we perform gene set enrichment analysis by selecting the top 10 genes with the highest velocities at each step of the interpolation process, with a focus on the first 40 steps (400 genes in total). Pathways related to morphogenesis, developmental process, and differentiation are significantly enriched, which demonstrates the ability of scVAEDer to detect related master regulators and pathways of this specific biological process (Fig. 5g).

## Discussion and conclusion

Analyzing scRNA-seq data is a challenging task which involves identifying the appropriate underlying data representation. The accuracy of this low-dimensional representation is crucial for many downstream tasks, including cell clustering, gene expression analysis, and visualization. By learning the correct representation of data, we are also able to generate novel samples, predict the effect of different perturbations on cells, and detect the master regulators of a gene regulatory network or marker genes of a biological process. To produce high-quality samples, the generative model should also be able to take into account and control both high-level and low-level variations between different cell types.

Here we propose scVAEDer, a novel method that unifies the power of VAE and DDM. scVAEDer can generate realistic gene expression data using the latent representations of VAE for high-level and DDM for low-level variations. scVAEDer has access to the lower dimension of data which enables us to predict the effect of different unseen perturbations on a variety of cell types. Using several datasets we show that scVAEDer outperforms the SOTA methods in predicting the effect of perturbations. Additionally, the DDM prior of our method can be used for interpolation between different cell types, allowing us to generate new cells along the differentiation trajectory and identify highly expressed genes in each step. We also demonstrate that scVAEDer’s ability to compute gene velocity (fast responder genes) along the interpolation path can be useful in cellular reprogramming. Previous studies have demonstrated that master regulators are not typically the genes with the highest differential expression [59]. To address this, we focus on genes with high velocities instead and are able to discover both novel and previously identified master regulators and pathways that have been shown to play a vital role in cell fate and cellular reprogramming. It would be beneficial to conduct additional experiments to determine whether these identified key genes are specific to the biological process studied here or if they hold importance across multiple distinct decision-making processes. Also, investigating the relationship between high expression values of these genes and their status as master regulators could provide deeper insights into their regulatory functions in various biological contexts. Unlike some generative models that rely mainly on autoencoders, scVAEDer leverages the VAE primarily for its efficient representation learning capabilities and DDM as the generative model. This approach enables scVAEDer to excel in downstream tasks without the limitations typically associated with autoencoder-based generative models such as prior hole.

Future versions of scVAEDer can explore novel approaches to enhance its performance. One potential avenue lies in reformulating the DDM component as a continuous process. This can be achieved through the implementation of a stochastic diffusion equation (SDE) or a deterministic approach like probability flow ordinary differential equations (ODEs). Moreover, scVAEDer’s architecture offers an opportunity for integration with other representation learning methods beyond VAE. Incorporating causal representation learning also holds promise, as it has the potential to not only capture the data’s intrinsic structure but also unveil the underlying causal relationships between key variables. These modifications can improve scVAEDer’s performance in future versions.

## Methods

### Variational autoencoder (VAE)

VAE is a generative model that is widely used in different deep learning applications. It can generate new samples by capturing the underlying data distribution. It is based on the idea of maximizing a lower bound of the log-likelihood of the data. This is achieved by minimizing the KL divergence between the learned distribution and the true distribution. VAE models typically consist of two parts: an encoder that transfers input data to a latent space and a decoder network that maps latent space back to the original data space. The objective function can be written as:

$$L\left(\theta,\phi;x\right)\;=\;-D_{KL}\;(q_\phi(z\vert x)\vert\vert p_\theta(z))\;+\;E_{q_\phi(z\vert x)}\;\lbrack log_{p_\phi}\;(x\vert z)\rbrack$$

where $\theta$ and $\phi$ are the parameters of the decoder and encoder networks, $x$ is the input data, $z$ is the latent variable, ${q}_{\phi }\left(z|x\right)$ is the approximate posterior distribution over the latent variables, and ${p}_{\theta }\left(x|z\right)$ is the conditional distribution over the input data given the latent variables. In the equation above the first term is the KL divergence between the approximate posterior and the prior distribution ${p}_{\theta }\left(z\right)$. That encourages the learned distribution to be as close as possible to the prior distribution. The second term is reconstruction loss, which measures how well the model can reconstruct the input data from the latent embedding. By minimizing this objective function, the VAE model can learn to generate new samples similar to the input data.

### Deep diffusion model (DDM)

Diffusion models belong to a category of generative models that have gained popularity in recent years. They can generate novel data from complex distributions, with higher quality compared to the existing models in this domain. To capture the underlying data distribution, DDMs follow a two-step process: a forward diffusion process and a reverse denoising process, working together in a coordinated manner. The forward process gradually destroys the structure of the data by adding noise, while the reverse process learns to recover the original data from the Gaussian noisy input.

It should be emphasized that the forward noising process is not trainable, but the reverse denoising process is parametrized and learned during training. In these processes, a first-order Markov chain with Gaussian transitions for the forward and backward processes is mostly used. The forward process can be written as:

$$q\left({\mathbf x}_t\vert{\mathbf x}_{t-1}\;\right)\;=\;N\left({\mathbf x}_t;\sqrt{1-\beta_t}{\mathbf x}_{t-1},\beta_t\mathbf I\right)$$

$$q\;({\mathbf x}_{1:T}\vert{\mathbf x}_0)\;=\;\prod_{t\;=\;1}^Tq\;({\mathbf x}_t\;\vert{\mathbf x}_{t-1})$$

By choosing the correct values of the noise scheduler, we can assume $q\left({x}_{t }|{x}_{0}\right)$ is isotropic Gaussian. By using the reparameterization trick, we get:

$$q\left(x_t\vert x_0\right)=N\left(x_t;\sqrt{\overline\alpha}x_0,\left(1-{\overline\alpha}_t\right)I\right)$$

where:

$$\alpha_t=1-\beta_t,$$

$${\overline\alpha}_t=\prod_{s=0}^t\alpha_s$$

It is worth mentioning that besides the Gaussian distribution, there are other distributions such as the gamma distribution that can be used. These distributions share a common property, which is the fact that the sum of two or more distributions results in the same distribution as well [60].

The reverse process can be formulated as:

$$p_\theta\left({\boldsymbol x}_{t-1}\vert{\boldsymbol x}_t\right)=\mathcal{N}\left({\boldsymbol x}_{t-1};{\boldsymbol\mu}_\theta\left({\boldsymbol x}_{\mathbf t},t\right),{\sum\limits}{\textstyle_\theta}\left({\boldsymbol x}_{\mathbf t},t\right)\right)$$

$$p_\theta\left(x_{0:T}\right)=p\left(x_T\right)\prod_{t=1}^Tp_\theta\left(x_{t-1}\vert x_t\right)$$

which starts from a random Gaussian $x_T:=\mathcal{N}\left(x_T,0,I\right)$, and in each step, the model learns ${p}_{\theta }\left({x}_{0:T}\right)$. To train the reverse process, it has been shown [21] that the loss function below works well as it leads to a better result:

$$L_{\mathrm{simple}}\left(\theta\right):= \mathbb{E}_{t,x_0,\boldsymbol{\epsilon}}\left[\parallel\boldsymbol{\epsilon}-{\boldsymbol{\epsilon}}_{\mathbf\theta}\left(\sqrt{{\overline\alpha}_{t}}{\mathbf x}_0+\sqrt{1-{\overline\alpha}_{t}}\boldsymbol{\epsilon},t\right)\parallel^2\right]$$

We can modify the above equation for our case as we train the diffusion model on the latent embedding of VAE. For the sake of simplicity, we ignore some of the weight terms [21]. As a result, we express the DDM part of the scVAEDer objective as follows:

$$L_{\mathrm{modified}}\left(\theta\right):=\mathbb{E}_{t,\boldsymbol z\sim q_\varnothing,\left(\boldsymbol z\vert\mathbf x\right),\;\boldsymbol{\epsilon}}\left[\parallel\boldsymbol{\epsilon}-{\boldsymbol{\epsilon}}_{\mathrm\theta}\left(\sqrt{{\overline\alpha}_{t}}{\boldsymbol z}_0+\sqrt{1-{\overline\alpha}_{t}}\boldsymbol{\epsilon},t\right)\parallel^2\right]$$

where $z$ is the encoded value of $x$ using the VAE’s encoder ${{q}_{\phi }}\left(z|x\right)$. Once the training process is complete, it is possible to sample from the DDM prior iteratively using the sampling method below:

$${\boldsymbol z}_t-1{\textstyle\frac1{\sqrt{\alpha_t}}}\left({\boldsymbol z}_t-{\textstyle\frac{1-\alpha_t}{\sqrt{1-{\overline\alpha}_t}}}{\boldsymbol\in}_\theta\left({\boldsymbol z}_t,t\right)\right)+\sigma_t\mathbf J$$

$${\boldsymbol J}_T\sim N\left(\mathbf0,\boldsymbol I\right)$$

### Interpolation

A number of interpolation settings have been considered in previous generative models to understand the trajectory between two states [22, 24, 29]. Here we have implemented two distinct interpolation approaches:

1. By leveraging the VAE, we encode ${x}_{1}$ and ${x}_{2}$ (gene values) to obtain ${z}_{1}$ and ${z}_{2}$, respectively, in the VAE’s latent embedding. Afterward, by using the diffusion forward process, we can obtain ${q}_{1}$ and ${q}_{2}$ in the prior space of DDMs. The interpolation can be performed in that space using ${\hat{\mathbf{q}}}_{12}\left(\lambda\right)=\left(1-\lambda\right){\hat{\mathbf{q}}}_1+\lambda{\hat{\mathbf{q}}}_2$ , where $\lambda$ is between [0,1]. Then the reverse process map ${\hat{\mathbf{q}}}_{12}$ back to the VAE’s latent space and then by using the decoder of the VAE we can compute the interpolation values in the gene space.
2. Following a similar approach as the previous part, we compute ${z}_{1}$ and ${z}_{2}$ using the VAE’s encoder, and subsequently conduct interpolation using ${\hat{\mathbf{z}}}_{12}\left(\lambda\right)=\left(1-\lambda\right){\hat{\mathbf{z}}}_1+\lambda{\hat{\mathbf{z}}}_2$. Then by adding Gaussian noise to ${\hat{\mathbf{z}}}_{12}$ , and performing the reverse diffusion process, we are able to generate better quality results than those produced solely by VAE. As in the previous section, the output of the reverse process is fed to the VAE’s decoder to map the embedding values from the VAE to the gene space.

The choice of interpolation approach is determined by the objective and the computational resources available. In this paper, we use the first approach for interpolation tasks as it provides greater flexibility and control over the number of diffusion steps and the amount of noise added. This level of control allows for a range of interpolation options, from coarse to fine adjustments, which can be advantageous in various scenarios. Previous studies have effectively demonstrated the benefits of this approach [21]. Alternatively, for users with limited computational resources, the second method offers a more straightforward and computationally efficient solution.

Also, to achieve more consistent results, different methods can be used, such as an implicit instead of a probabilistic method (DDIM over DDPM) or the deterministic probability flow ODEs [22, 61]. Here we fix the stochasticity for all generated samples during interpolation to increase smoothness between samples. It is also essential to select the appropriate number of diffusion steps for each unique task, as this determines whether interpolation takes place at more refined or coarse levels of detail [21]. When the diffusion step is zero, interpolation takes place in the VAE’s latent space. As we increase the number of diffusion steps, the initial interpolation information gradually diminishes, resulting in interpolations that are less detailed, coarser, and more varied (Additional file 1: Fig S2).

### Model architecture and training process

scVAEDer is trained on 4 Nvidia Tesla M60 machines with 32 GB GPUs, 448 GB system RAM, and 48 vCPUs. The training process involves two stages (detailed in Additional file 1: Tables S2 and S3). The initial stage trains a VAE model, followed by the second stage where the diffusion model uses the latent embedding learned from the trained VAE. We choose a two-stage training process due to the higher memory and resource requirements of end-to-end training. Also, previous research has shown that end-to-end training can reduce both the quality of the generated data and the performance of the model [24]. To ensure that the model can accurately learn the data embedding and generate high-quality samples, it is essential to select suitable parameters, such as the VAE and DDM network architectures, the number of diffusion steps, and the level of noise added during the forward process (Additional file 1: Tables S2 and S3). Therefore, a random hyperparameter search is performed on these parameters to minimize the loss functions, ultimately leading to better model performance. Notably, high-quality data generation in diffusion models depends on a crucial factor: the interplay between the number of steps and layers within the DDMs. While intuitively increasing these parameters seems beneficial, a critical trade-off exists. On the one hand, a larger number of steps facilitates a more gradual and controlled noise manipulation process. This finer control can lead to potentially higher fidelity in the generated data. However, this benefit comes at a significant cost. A substantial increase in computational resources is required as the number of steps and layers grows. Furthermore, excessively high step counts can lead to overfitting, resulting in unrealistic or overly smooth outputs that lack the natural characteristics of real data. Therefore, achieving a balance between computational complexity and quality of data generation is crucial. This can be achieved based on the available computational resources and checking the performance of the model with different hyperparameters to avoid overfitting and ensure optimal data generation. We observed that identifying the optimal DDM parameters, such as the number of diffusion steps, plays a key role in increasing model performance, as it directly influences the quality of generated data.

### Exponential moving average (EMA)

EMA can be used to improve the performance of the training process. Unlike the conventional methods which directly adjust model weights, EMA calculates a running average of the model’s weight parameters over the training process. This can lead to a more stable set of parameters. In our case, the weight parameters for the latent DDMs are tracked using EMA with a decay value of 0.999.

### Variance scheduler

The noise schedules ${\beta }_{1}$ to ${\beta }_{T}$ are chosen such that the chain approximately converges to a standard Gaussian distribution after T steps. The variance parameter can be fixed to a constant or chosen as a schedule over the timesteps. To improve the performance, we incorporated three distinct variance schedulers, such as linear, quadratic, and sigmoidal. The user has the option to select any of these schedulers. The beta values are between $1{e}^{-5}$ and $0.5{e}^{-2}$ (Additional file 1: Fig. S4).

### For visualization, reduced dimension data

Clusters are visualized using the Python package “UMAP” [62].

### Data preprocessing

The scRNA-seq gene expression data are centered and log-normalized. 2000 highly variable genes were chosen. We also performed batch normalization on the latent layer of VAE before training DDM as we found it improves the convergence speed and accuracy of the model.

### Comparison with other methods

In comparison with alternative methods, we obtain the scGen python package from the GitHub repository https://github.com/theislab/scgen and configure the requisite experimental environment accordingly. Subsequent modifications are applied to the scGen architecture to reduce computational complexity. To ensure a consistent evaluation, we maintain identical neural network architecture and hyperparameters for both scVAEDer and scGen. For scPreGAN, we use the python package available at https://github.com/JaneJiayiDong/scPreGAN.git and follow the default parameters outlined in their publication, with the exception of adjusting the model to accommodate 256 nodes instead of 512 and 128 nodes instead of 256 to facilitate a fair comparison and alleviate computational cost. We also evaluated scVAEDer’s performance against VEGA [63]. We excluded VEGA’s performance from the comparison since its correlation values (ranging from 0.53 to 0.6 across cell types) were lower than those of other methods, such as scGen and scPreGAN. VEGA places a stronger emphasis on interpretability within its latent layer to provide insights into diverse biological experiments. The VEGA Python package was obtained from its GitHub repository: https://github.com/LucasESBS/vega.

## Supplementary Information

Additional file 1: Supplementary Figures. This file provides additional details and results from our study, including Figs. S1-S4. Supplementary Tables. This file provides additional details and results from our study, including Tables. S1-S3.

Additional file 2: Review History. This file contains the review history

## Data Availability

All datasets analyzed in the current study are publicly available and can be downloaded from their public repository. The zebrafish hematopoiesis [64] raw data can be found under the accession number E-MTAB-5530 on ArrayExpress. The raw PBMC of lupus patients [40] and Salmonella infection [65] datasets are available on the NCBI’s Gene Expression Omnibus under accession numbers GSE96583 and GSE92332. Raw reprogramming data [66] is available from the Gene Expression Omnibus under accession number GSE99915. The preprocessed version can be downloaded from [67]. Our Python implementation along with the versions of the packages used can be found at: https://github.com/MehrshadSD/scVAEDer [68] and 10.5281/zenodo.14834911 [69] under a Creative Commons Attribution 4.0 International license.
