# Supplementary material for: scVAEDer: integrating deep diffusion models and variational autoencoders for single-cell transcriptomics analysis
Source: Genome Biol. 2025 Mar 21;26:64. doi: 10.1186/s13059-025-03519-4 (PMC11927372; doi:10.1186/s13059-025-03519-4)
Supplement: Supplementary file 1 — Additional file 1: Supplementary Figures. This file provides additional details and results from our study, including Figs. S1-S4. Supplementary Tables. This file provides additional details and results from our study, including Tables. S1-S3. [file 13059_2025_3519_MOESM1_ESM.docx]

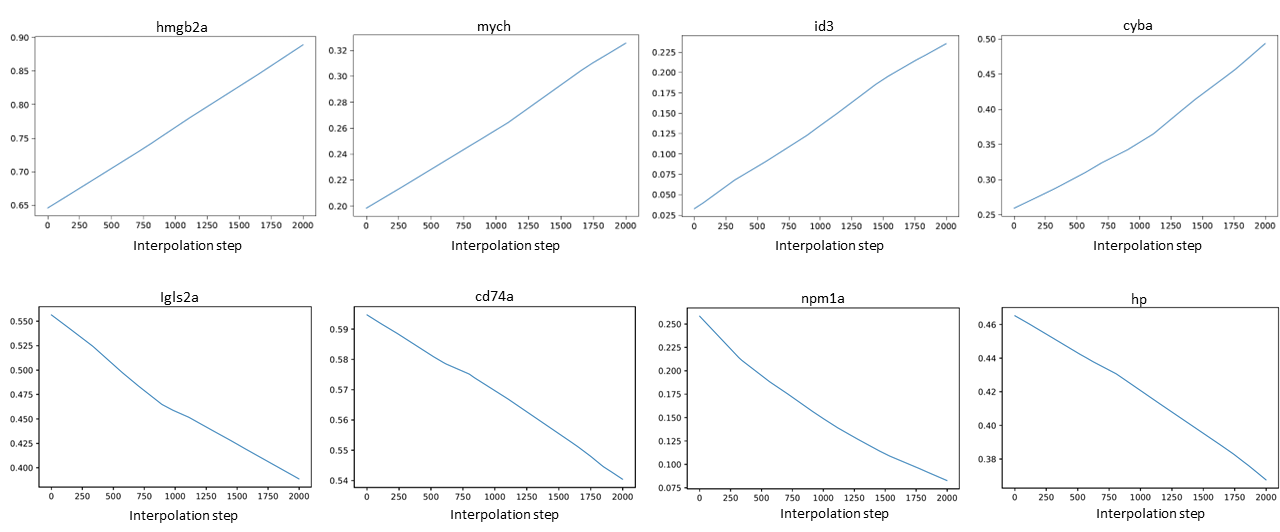


**Fig S1: Gene expression changes as a result of interpolation.** The expression of selected maker genes is upregulated or downregulated as we interpolate between monocytes to HSPCs with 2000 equidistant steps.


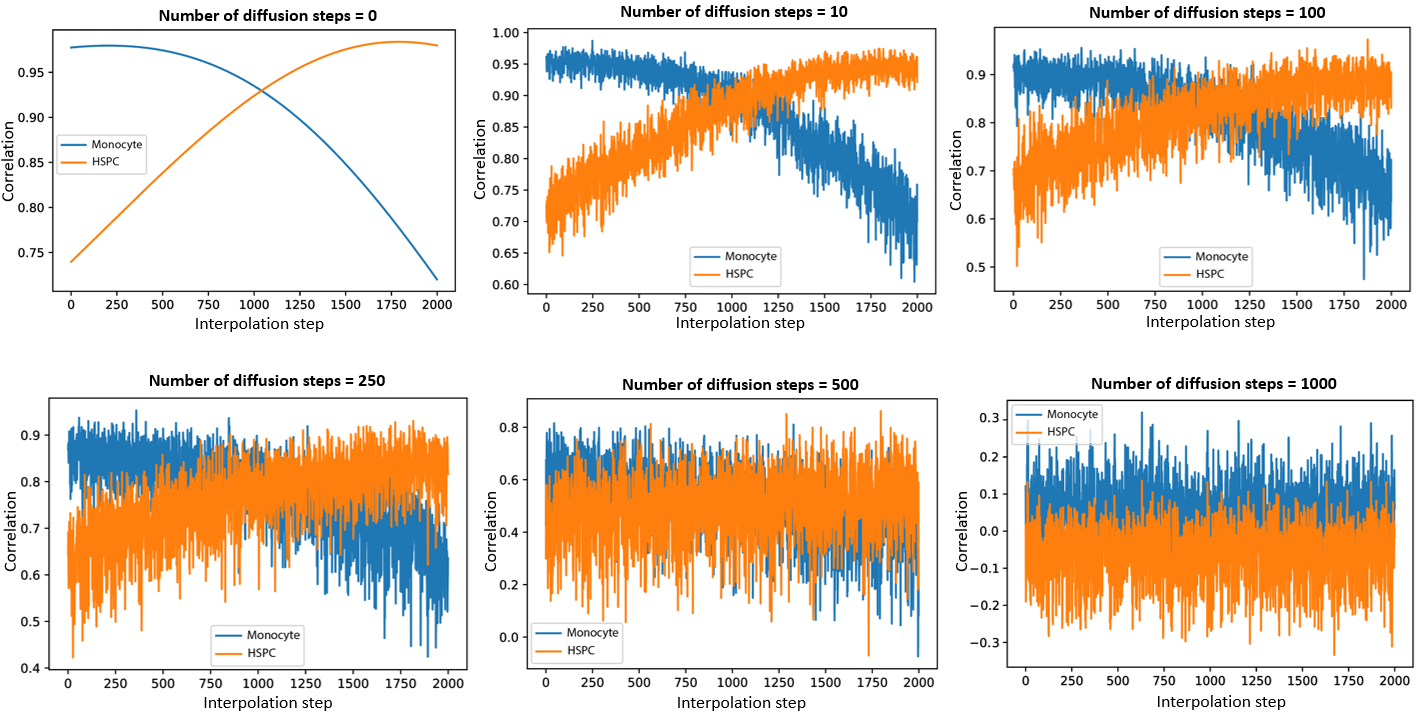


**Fig S2: The number of diffusion steps changes the level of detail in generated data.** Samples with different levels of granularity are generated by adjusting the number of diffusion steps before interpolation. If a large number of diffusion steps are used, more structure in the initial data will be destroyed, causing a gradual loss of information, resulting in coarser interpolations, and potentially causing abrupt changes in the correlation values between successive steps.


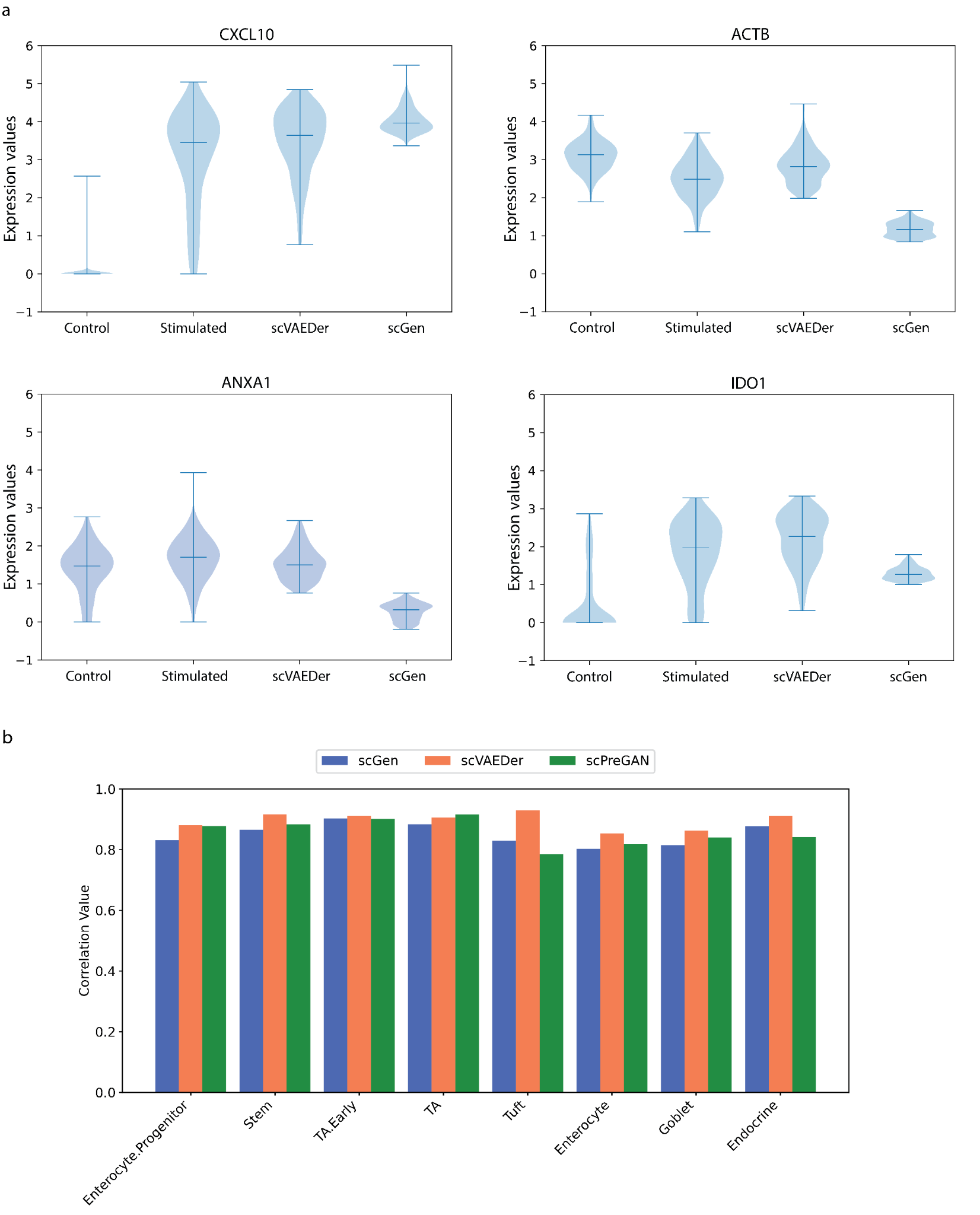


**Fig S3: scVAEDer can correctly predict the distribution of genes in the stimulated condition.** a, Violin plots showing the distribution of specific key response genes across control, real stimulated condition, and prediction of stimulation using scVAEDer and scGen in Dendritic cells (DC). scVAEDer more accurately predicts the effect of the perturbation on genes. b, Comparison of the correlation values of various cell types obtained using scGen, scPreGAN and scVAEDer for intestinal epithelial cells after Salmonella infections data.


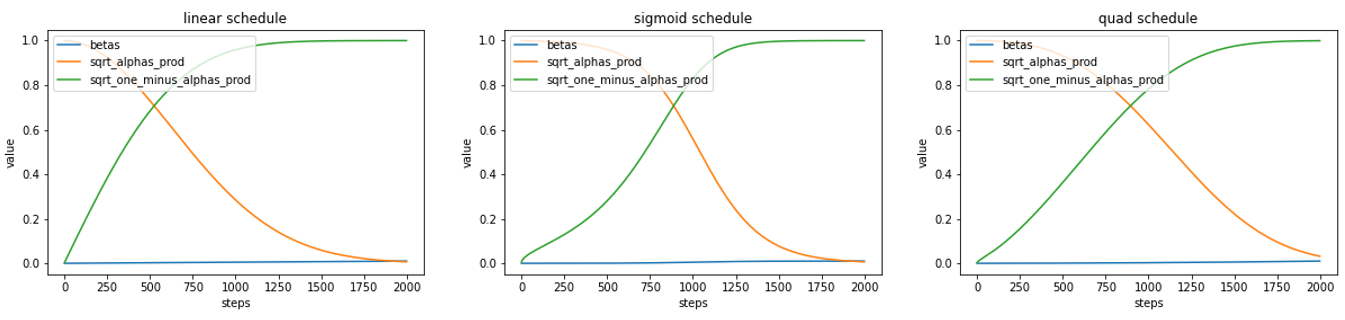


**Fig S4: DDM training and variance scheduling.** A comparison between the effect of different variance scheduling methods on $\alpha$ and $\beta$ values. The user can choose between one of these 3 methods prior to training the model.

| **Rank** | **Gene candidate** | **Gene Function** | **References** |
| --- | --- | --- | --- |
| 1 | Ptma | *Ptma* is involved in regulating the exit from pluripotency and its repression resulted in the strongest deviation from pluripotency. | [(1)](https://sciwheel.com/work/citation?ids=807339&pre=&suf=&sa=0) |
| 2 | Wnt4 | Loss of *Wnt4* induces the activation of stem cells and accelerates muscle regeneration. | [(2)](https://sciwheel.com/work/citation?ids=11167422&pre=&suf=&sa=0) |
| 3 | Kng1 | Reprogrammed iEP marker gene. | [(3)](https://sciwheel.com/work/citation?ids=14141248&pre=&suf=&sa=0) |
| 4 | Igf2 | *Igf2* deficiency increases HSC self-renewal and differentiation. | [*(4)*](https://sciwheel.com/work/citation?ids=14631812&pre=&suf=&sa=0) |
| 5 | Ctgf | *Ctgf* is a reliable factor to differentiate iPSCs efficiently. | [(5)](https://sciwheel.com/work/citation?ids=3586261&pre=&suf=&sa=0) |
| 6 | H2afx | H2afx is capable of controlling human stem cell self-renewal and lineage-specific differentiation. | [(6)](https://sciwheel.com/work/citation?ids=10928586&pre=&suf=&sa=0) |
| 7 | FoxA1 | It is known as the master regulator of the epigenome and fate. | [(7)](https://sciwheel.com/work/citation?ids=12637044&pre=&suf=&sa=0) |
| 8 | Apoa1 | Reprogramming initiation markers. | [(3)](https://sciwheel.com/work/citation?ids=14141248&pre=&suf=&sa=0) |
| 9 | Timp3 | *In vivo* analysis underpins its role as a master regulator of ectodomain shedding. | [(8)](https://sciwheel.com/work/citation?ids=14631838&pre=&suf=&sa=0) |
| 10 | Mt1 and 2 | Zinc maintains embryonic stem cell pluripotency | [(9)](https://sciwheel.com/work/citation?ids=8144202&pre=&suf=&sa=0) |
| 11 | H19 | Downregulation of *H19* improves the differentiation potential of mouse embryonic stem cells. | [(10)](https://sciwheel.com/work/citation?ids=14631816&pre=&suf=&sa=0) |
| 12 | Top2a | It is uniquely expressed in HSPCs and is important in the survival of human pluripotent stem cells. | [(11)](https://sciwheel.com/work/citation?ids=11423228&pre=&suf=&sa=0) |
| 13 | Igfbp | The Igfbp family is crucial for fibroblasts to progenitor cell dedifferentiation. | [(12)](https://sciwheel.com/work/citation?ids=11839691&pre=&suf=&sa=0) |
| 14 | Malat1 | Regulates myogenic differentiation and muscle regeneration. | [(13)](https://sciwheel.com/work/citation?ids=14631823&pre=&suf=&sa=0) |
| 15 | Lgals1 | Lgals1 is a transcriptional target of STAT3 and promotes mesenchymal glioblastoma. | [(14)](https://sciwheel.com/work/citation?ids=11845240&pre=&suf=&sa=0) |

**Table S1**: Top 15 genes ranked based on their velocity values, along with references that demonstrate their association with stem cell function and cellular reprogramming.

| **Name** | **Type** | **Size_zebrafish** | **Activation_**  **zebrafish** | **Size_**  **reprogramming**  **&**  **IFN-β** | **Activation_**  **reprogramming**  **&**  **IFN-β** |
| --- | --- | --- | --- | --- | --- |
| Input | … | Number of highly variable genes | … | Number of highly variable genes | … |
| Layer 2 | Fully connected | 150 | ReLU | 250 | ReLU and Dropout=0.15 |
| Mean | Fully connected | 30 | Linear | 45 | Linear |
| Variance | Fully connected | 30 | Linear | 45 | Linear |
| Sampling | Fully connected | 30 | Linear | 45 | Linear |
| Layer 4 | Fully connected | 150 | ReLU and Dropout=0.2 | 250 | ReLU |
| Output | Fully connected | Number of highly variable genes | ReLU | Number of highly variable genes | ReLU |
| - Optimization: Optimizer: Adam, Learning rate: 0.001, batch_size: 400, epochs: 250 - Number of highly variable genes: 2000 for all datasets (except for zebrafish hematopoiesis, for which 1845 genes are selected)   Hyperparameter search:  Layer size: for Zebrafish [200, 150, 100], for other datasets [300, 250, 200] /  Latent_layer: [15, 30, 45, 60] / Dropout: [0.15, 0.4] | | | | | |

**Table S2 -** Detailed architecture of VAE part of scVAEDer.

| **Name** | **Type** | **Size_**  **zebrafish** | **Activation_**  **zebrafish** | **Size_**  **reprogramming**  **&**  **IFN-β** | **Activation_**  **reprogramming**  **&**  **IFN-β** |
| --- | --- | --- | --- | --- | --- |
| Input | … | Size of latent layer | … | Size of latent layer | … |
| Layer1 | Fully connected | 512 | ReLU | 1200 | ReLU |
| Layer 2 | Fully connected | 512 | ReLU | 1200 | ReLU |
| Layer 3 | Fully connected | 512 | ReLU | 1200 | ReLU |
| Output | Fully connected | Size of latent layer | … | Size of latent layer | … |
| - Optimization_Zebrafish: Optimizer: Adam, Learning rate: 0.001, batch_size: 1500, epochs: 4000 - Optimization_Reprogramming: Optimizer: Adam, Learning rate: 0.001, batch_size: 1500, epochs: 4000 - Optimization_IFN-β: Optimizer: Adam, Learning rate: 0.001, batch_size: 10000, epochs: 4000   Hyperparameter search:  Layer size: for Zebrafish [512, 256, 128, 64], for other datasets [1200,1000,800,600,400,100] /  Number of steps: [500, 750, 1000, 1250, 1500, 2000] / Noise schedule functions: [Linear, Quadratic, Sigmoid] / beta_start:[1e-5, 1e-4], beta_end:[1e-2, 5e-2] | | | | | |

**Table S3 -** Detailed architecture of DDM part of scVAEDer.

**References**

[1. Kolodziejczyk AA, Kim JK, Tsang JCH, Ilicic T, Henriksson J, Natarajan KN, et al. Single Cell RNA-Sequencing of Pluripotent States Unlocks Modular Transcriptional Variation. Cell Stem Cell. 2015 Oct 1;17(4):471–85.](https://sciwheel.com/work/bibliography/807339)

[2. Wang C, Rabadan Ros R, Martinez-Redondo P, Ma Z, Shi L, Xue Y, et al. In vivo partial reprogramming of myofibers promotes muscle regeneration by remodeling the stem cell niche. Nat Commun. 2021 May 25;12(1):3094.](https://sciwheel.com/work/bibliography/11167422)

[3. Kamimoto K, Adil MT, Jindal K, Hoffmann CM, Kong W, Yang X, et al. Gene regulatory network reconfiguration in direct lineage reprogramming. Stem Cell Reports. 2023 Jan 10;18(1):97–112.](https://sciwheel.com/work/bibliography/14141248)

[4. Barroca V, Lewandowski D, Jaracz-Ros A, Hardouin S-N. Paternal Insulin-like Growth Factor 2 (Igf2) Regulates Stem Cell Activity During Adulthood. EBioMedicine. 2017 Feb;15:150–62.](https://sciwheel.com/work/bibliography/14631812)

[5. Songstad AE, Worthington KS, Chirco KR, Giacalone JC, Whitmore SS, Anfinson KR, et al. Connective Tissue Growth Factor Promotes Efficient Generation of Human Induced Pluripotent Stem Cell-Derived Choroidal Endothelium. Stem Cells Transl Med. 2017 Jun;6(6):1533–46.](https://sciwheel.com/work/bibliography/3586261)

[6. Orlando L, Tanasijevic B, Nakanishi M, Reid JC, García-Rodríguez JL, Chauhan KD, et al. Phosphorylation state of the histone variant H2A.X controls human stem and progenitor cell fate decisions. Cell Rep. 2021 Mar 9;34(10):108818.](https://sciwheel.com/work/bibliography/10928586)

[7. Balsalobre A, Drouin J. Pioneer factors as master regulators of the epigenome and cell fate. Nat Rev Mol Cell Biol. 2022 Jul;23(7):449–64.](https://sciwheel.com/work/bibliography/12637044)

[8. Spanò DP, Scilabra SD. Tissue Inhibitor of Metalloproteases 3 (TIMP-3): In Vivo Analysis Underpins Its Role as a Master Regulator of Ectodomain Shedding. Membranes (Basel). 2022 Feb 11;12(2).](https://sciwheel.com/work/bibliography/14631838)

[9. Mnatsakanyan H, Sabater I Serra R, Salmeron-Sanchez M, Rico P. Zinc maintains embryonic stem cell pluripotency and multilineage differentiation potential via AKT activation. Front Cell Dev Biol. 2019 Aug 30;7:180.](https://sciwheel.com/work/bibliography/8144202)

[10. Ragina NP, Schlosser K, Knott JG, Senagore PK, Swiatek PJ, Chang EA, et al. Downregulation of H19 improves the differentiation potential of mouse parthenogenetic embryonic stem cells. Stem Cells Dev. 2012 May 1;21(7):1134–44.](https://sciwheel.com/work/bibliography/14631816)

[11. Desai RV, Chen X, Martin B, Chaturvedi S, Hwang DW, Li W, et al. A DNA repair pathway can regulate transcriptional noise to promote cell fate transitions. Science. 2021 Aug 20;373(6557).](https://sciwheel.com/work/bibliography/11423228)

[12. Chen P-C, Kuo Y-C, Chuong C-M, Huang Y-H. Niche Modulation of IGF-1R Signaling: Its Role in Stem Cell Pluripotency, Cancer Reprogramming, and Therapeutic Applications. Front Cell Dev Biol. 2020;8:625943.](https://sciwheel.com/work/bibliography/11839691)

[13. Chen X, He L, Zhao Y, Li Y, Zhang S, Sun K, et al. Malat1 regulates myogenic differentiation and muscle regeneration through modulating MyoD transcriptional activity. Cell Discov. 2017 Mar 14;3:17002.](https://sciwheel.com/work/bibliography/14631823)

[14. Sharanek A, Burban A, Hernandez-Corchado A, Madrigal A, Fatakdawala I, Najafabadi HS, et al. Transcriptional control of brain tumor stem cells by a carbohydrate binding protein. Cell Rep. 2021 Aug 31;36(9):109647.](https://sciwheel.com/work/bibliography/11845240)
