# Supplementary material for: scVAEDer: integrating deep diffusion models and variational autoencoders for single-cell transcriptomics analysis
Source: Genome Biol. 2025 Mar 21;26:64. doi: 10.1186/s13059-025-03519-4 (PMC11927372; doi:10.1186/s13059-025-03519-4)
Supplement: Supplementary file 2 — Additional file 2: Review History. This file contains the review history [file 13059_2025_3519_MOESM2_ESM.docx]

**1^st^ round**

**Reviewer 1**

In this manuscript, Sadria et al. proposed a generative model, named scVAEDer, which integrates deep diffusion models (DDM) and variational autoencoders (VAE) to capture the pattern of latent embeddings of single-cell RNA sequencing (scRNA-seq) data. Through experiments on multiple datasets, they claimed that scVAEDer showed superior performance in generating scRNA-seq data, estimating intermediate cell states and predicting gene expression changes during the transition between cells, predicting perturbation responses and identifying master regulatory genes. The idea of combining VAE and DDM to avoid the 'prior hole' problem is interesting. However, the overall writing logic of the Methods section of the manuscript is relatively disorderly, making it difficult for readers to follow. Besides, the persuasiveness of the results section needs to be strengthened due to insufficient comparison with existing methods and lack of necessary details.

Major comments:
1. There are a lot of garbled formulas in the manuscript which makes it really hard to follow.
2. The authors claimed that scAVEDer learns a meaningful representation which can capture both global semantics and local variations in the data. Low-dimensional visualization, clustering and other downstream analyses like trajectory inference are common approaches to demonstrate the representation ability of dimension reduction methods. However, there are no such experiments to illustrate the performance of scAVEDer.
3. According to Methods section, "The initial stage trains a VAE model, followed by the second stage where the diffusion model uses the latent embedding learned from the trained VAE". I am wondering what is the latent embedding of the trained VAE model? Is it the mean vector of the encoder of VAE model? Besides, the final embedding of scVAEDer also seems not to be clearly indicated. The input and output of the model should be pointed out since this is important for readers to understand the model.
4. The authors claimed that "VAEs can experience posterior collapse in which the learned mapping reduces significant portions of the input data to a single point in the output" in Introduction section. However, the authors still utilized VAEs as the first step of their model and train the diffusion model with the latent embedding learned from the trained VAE, as described in "We chose a two-stage training process in which the diffusion model uses the latent embedding learned from the trained VAE in the second stage" of Methods section. Therefore, I am curious how their model solves the "prior holes" problem since the training of VAE is the first step of their model?
5. The authors described two distinct interpolation approaches in Methods section and illustrated the modeling of transition between cells in Figs. 3 of Results section. However, the author did not seem to indicate in the manuscript which interpolation approach was used to obtain such results. Besides, what is the difference in performance of the two approaches? The authors should provide more comparative experiments and guidance on the selection of these two approaches.
6. The authors' demonstration of the experimental efficacy of scVAEDer in perturbation prediction requires further scrutiny. scGen (Lotfollahi et al., 2019), claimed to be SOTA, is already a relatively early method, and subsequent methods such as scPreGAN (Wei et al., Bioinformatics 2022), scVIDR(Kana et al., Patterns 2023), CellOT(Bunne et al., Nature methods 2023), etc., have been ignored.
7. According to the scGen tutorial, it is observed that scGen achieves determination coefficient of mean genes expression in various cell types in the PBMC dataset, mostly above 0.9, even reaching 0.95. However, the results reproduced by the author in Figure 4b appear significantly lower. Did the authors calculate the regression coefficients in Figure 4b using all genes or only a subset of differentially expressed genes (DEGs)?
8. As Figures 4c and 4d do not annotate additional information such as DEGs like scGen, Figure 4b actually encompasses the information present in Figures 4c and 4d. I would recommend the authors utilize low-dimensional visualization to present more details of more cell types.
9. It is not clear how the genes were selected for Figure 4e, and the origin of the gene data concerning which cell type is not specified. Additionally, the crucial ISG15 gene, pivotal in perturbation, has not been subjected to comparison.
Minor points:
1. Typos and tenses should be double checked, such as "we train(ed) new models and showed the stimulation" in Line 23 of Page 10 and "Then the reverse process map(s)" in Lines 17-18 of Page 17.

**Reviewer 2**

In this manuscript, the authors proposed a novel framework, scVAEDer, to learn a representation which can capture both global semantics and local variations. They overcome the limitations of current generative models (VAES, GANs) by employing a Denoising Diffusion Models (DDMs) and combine the strengths of VAEs and DDMs. Then it can be used for multiple downstream tasks, such as data generation, cell differentiation, prediction of gene expression and detection of master regulators. They tested the model on different datasets and demonstrated that scVAEDer outperformed most competing methods across most datasets. However, the results from the conducted experiments are not sufficient and compelling, which fail to demonstrate the significance of scVAEDer and its advantages over the existing methods. Detailed problems are given as follows.
Major
1.
The authors only provided a case analysis 'interpolate between monocytes to Hematopoietic Stem/Progenitor Cells (HSPC)' in the part of scVAEDer models the transition between cells.
I suggest that the authors provide references or a detailed explanation as to why this specific cell transition (from monocytes to Hematopoietic Stem/Progenitor Cells) was chosen as the study case. If the aim of the article is to demonstrate the general applicability of the scVAEDer method, including a broader range of cell transitions might make the argument more convincing.
2.
The authors only provided limited evidence to show the advantages of the proposed model over the state-of-the-art methods.
In all downstream analyses, apart from the section 'scVAEDer predicts perturbation responses using feature manipulation', there is no comparison with state-of-the-art (SOTA) methods in the other sections. Moreover, this analysis only chose one method, scGen, and a single dataset (PBMCs) for comparison, does not provide substantial evidence to assert that scVAEDer is superior to other methods. Additionally, are there limitations in the types of single-cell transcriptomics data it can handle? If not, why is each experiment conducted using only one dataset? Please give more description.
3.
The authors only provided an analysis of the data generation capabilities of VAEs and DDMs, but the absence of any ablation studies in the paper.
This manuscript lack of the ability to validate the effectiveness of these two components within the model. For instance, it remains unclear how the results would vary if only the DDM model were used, or if a module with similar functionality were to replace the VAE. This lack of empirical evidence makes it challenging to conclusively demonstrate that the integration of both VAE and DDM is essential for the model's performance.
4.
In the "Model Architecture and Training Process" section, the authors fail to provide sufficient details about the model, raising concerns about the replicability of the experiment.
Firstly, there is a lack of specific details regarding the hyperparameter search, such as the search space, algorithms used, and evaluation criteria, all of which are crucial for assessing the model's performance and reproducibility. Secondly, while the trade-off between performance and computational resources is mentioned, the authors do not elaborate on how this balance was achieved, nor do they discuss how it impacts the final performance of the model.
5.
The article does not include extensive validation of the scVAEDer model using benchmark datasets or real biological data, raising concerns about its generalizability and reliability.
Minor
1.
Regarding Fig.1, the unclear workflow presented can easily lead to confusion for readers. For instance, it is not apparent whether the training proceeds from top to bottom or from left to right. Clarifications are needed on where the data input is located, and which variable units interact between the VAE and DDM.
2.
There is a discrepancy between the textual description and the figure representation. The text mentions that 𝑍𝑠𝑒𝑚 is used to train a diffusion model, yet Fig.1 illustrates X. This inconsistency needs to be addressed to ensure coherence and understanding.
3.
In the "Data Availability" section, there is no mention of the PBMC dataset used in the part of the study where scVAEDer predicts perturbation responses through feature manipulation.
4.
It is recommended that the authors present statistical results from multiple experiments to validate the robustness of the model.
5.
Fig.5(g) is not cited in the main text, and it lacks a detailed biological interpretation and relevance of its results. Providing examples of novel biological discoveries or confirming known phenomena would significantly strengthen the manuscript.

**Authors’ response**

We appreciate the editor and reviewers for their detailed feedback on our manuscript. Their insightful comments have played a crucial role in refining our work, improving clarity, and ensuring its completeness. We truly value their positive and constructive responses.

In response to their feedback, we have thoroughly addressed each comment as outlined below. Additionally, we have incorporated the necessary modifications into the manuscript, which are presented in a marked-up form to highlight the changes made. Also, to improve the robustness and clarity of our manuscript, we have introduced a new dataset and made adjustments to the figures. We hope the revised manuscript meets the high standards of Genome Biology. Once again, we thank the editor and reviewers for their invaluable input, which has greatly helped us improve our work.

**Reviewer #1:** In this manuscript, Sadria et al. proposed a generative model, named scVAEDer, which integrates deep diffusion models (DDM) and variational autoencoders (VAE) to capture the pattern of latent embeddings of single-cell RNA sequencing (scRNA-seq) data. Through experiments on multiple datasets, they claimed that scVAEDer showed superior performance in generating scRNA-seq data, estimating intermediate cell states and predicting gene expression changes during the transition between cells, predicting perturbation responses and identifying master regulatory genes. The idea of combining VAE and DDM to avoid the 'prior hole' problem is interesting. However, the overall writing logic of the Methods section of the manuscript is relatively disorderly, making it difficult for readers to follow. Besides, the persuasiveness of the results section needs to be strengthened due to insufficient comparison with existing methods and lack of necessary details.

**Major comments:**

1. There are a lot of garbled formulas in the manuscript which makes it really hard to follow.

We apologize for any inconvenience caused by the unreadable formulas in the manuscript. This issue is likely due to a conversion error during the PDF generation process. To ensure the clarity and readability of our manuscript, we submit both the PDF and the original editable format (Word document) for your consideration.

2. The authors claimed that scAVEDer learns a meaningful representation which can capture both global semantics and local variations in the data. Low-dimensional visualization, clustering and other downstream analyses like trajectory inference are common approaches to demonstrate the representation ability of dimension reduction methods. However, there are no such experiments to illustrate the performance of scAVEDer.

Thank you for this insightful comment. While scVAEDer shares some similarities with dimension reduction methods, it primarily operates as a generative model due to its combined variational autoencoders (VAE) and deep diffusion model (DDM) architectures. This allows us to generate novel and realistic single-cell RNA-seq data, and perform various downstream predictive tasks with high accuracies, such as identifying perturbation responses on different cell types and predicting gene expression changes in different cellular transitions.

Recognizing the importance of clustering and its implications for subsequent analyses in attaining a meaningful learned representation,

1. We have demonstrated the ability of the VAE component in scVAEDer to accurately separate different cell types (clusters) and conserve the data structure within the latent representation using various biological datasets with different sizes (Figures 2a, 4a, 5a). This suggests that the learned representation captures meaningful information from the data.

2. The DDM component of scVAEDer, enables us to generate realistic gene expression profiles for all cell types from just Gaussian noise through the reverse process. These generated cells also clearly separate into distinct cell types (clusters), and show a high gene expression similarity to the real data (figure 2c,e as an example), further showing the ability of scVAEDer to capture variations in the generated data.

Moreover, to further test the model on other downstream tasks, we demonstrated scVAEDer's ability to use the learned representation to:

1. Accurately predict perturbation responses for unseen cell types, which shows the generalizability of the learned representation beyond the training data (figure 4).

2. Identify relevant known and novel biological pathways and key genes for specific processes, highlighting the ability of scVAEDer to extract meaningful biological signals from the learned representation (figure 5e,g).

The performance of scVAEDer across these downstream tasks indicates it learns a meaningful representation that accounts for both local and global variations within the data. This aligns with findings in other deep learning areas, where latent diffusion models (VAEs +DDMs) have proven effective for capturing both broad patterns and specific variations within data [(1–4)](https://sciwheel.com/work/citation?ids=13075114,14630581,14630596,13328793&pre=&pre=&pre=&pre=&suf=&suf=&suf=&suf=&sa=0,0,0,0). To further support this aspect of our model and provide a clearer explanation, we have included additional descriptive details in the text and incorporated relevant citations for the reader's reference.

3. According to Methods section, "The initial stage trains a VAE model, followed by the second stage where the diffusion model uses the latent embedding learned from the trained VAE". I am wondering what is the latent embedding of the trained VAE model? Is it the mean vector of the encoder of VAE model? Besides, the final embedding of scVAEDer also seems not to be clearly indicated. The input and output of the model should be pointed out since this is important for readers to understand the model.

We appreciate your insightful feedback. To clarify the process:

During training, scVAEDer initially trains a VAE with a standard Gaussian prior using the single-cell gene expression data as input. The latent embedding, in this context, is essentially a compressed representation of the input data in a lower-dimensional form. Subsequently, scVAEDer extracts latent embeddings z of scRNA-seq data using the encoder part of the VAE q(z|x) and then fits the diffusion model to the latent space formed by the output of the encoder (Figure 1). This training procedure involves progressively adding Gaussian noise into the learned latent embedding and aims to learn how to reverse the noise addition through the reverse diffusion process (Figure 1). Our diffusion model learns how to effectively map all states into its own Gaussian prior. The objective function guiding this training process is detailed in Methods.

To clarify the process, Figure 1 has been modified to better depict the scVAEDer architecture, pipeline, input, output, and various components, including the distinct training stages for the VAE and DDM. Additionally, we have provided a comprehensive explanation within the text and the figure caption to facilitate a smoother understanding of the model's flow.

4. The authors claimed that "VAEs can experience posterior collapse in which the learned mapping reduces significant portions of the input data to a single point in the output" in Introduction section. However, the authors still utilized VAEs as the first step of their model and train the diffusion model with the latent embedding learned from the trained VAE, as described in "We chose a two-stage training process in which the diffusion model uses the latent embedding learned from the trained VAE in the second stage" of Methods section. Therefore, I am curious how their model solves the "prior holes" problem since the training of VAE is the first step of their model?

Indeed, standard VAEs with a Gaussian prior often struggle to consider the complexity and multimodality of the true data distribution in the latent space, leading to the well-known "prior hole" problem. This limitation restricts the VAE's effectiveness as a generative model for accurately simulating meaningful data from its latent representation. Our work acknowledges this limitation, and we emphasize the importance of using VAE primarily as an efficient and fast reconstruction and dimensionality reduction method, rather than relying on them as generative models. Figure 2 demonstrates this limitation. Samples drawn from the VAE's Gaussian prior fail to accurately reproduce the realistic and complex distribution of the actual data. This discrepancy underscores the limitation of VAEs as generative models, in accurately generating the complex distribution of single-cell gene expression data.

Furthermore, we recognize that the "prior hole" problem can present itself during transitions between cell types and states when interpolating between their corresponding encodings in the VAE’s latent space. Direct interpolation in the latent space between different VAE encodings might inadvertently pass through "empty" regions, leading to the generation of artifacts or unrealistic representations [(5)](https://sciwheel.com/work/citation?ids=14630616&pre=&suf=&sa=0).

To overcome these limitations, scVAEDer leverages the strengths of both VAEs and diffusion models in a complementary way. We use the VAE's efficient reconstruction capabilities to learn a meaningful and low-dimensional representation of the data and then employ the DDM as a generative model for simulating realistic gene expression data. Additionally, we perform interpolations within the DDM's latent space instead of the VAE's. This approach avoids potential artifacts that might arise from traversing "empty" regions in the VAE's space during transitions between cell types and states.

We have now included an additional paragraph in the discussion and explained it in more detail.

5. The authors described two distinct interpolation approaches in Methods section and illustrated the modeling of transition between cells in Figs. 3 of Results section. However, the author did not seem to indicate in the manuscript which interpolation approach was used to obtain such results. Besides, what is the difference in performance of the two approaches? The authors should provide more comparative experiments and guidance on the selection of these two approaches.

The choice of interpolation method depends on the objective and available computational resources of each user. The first approach offers greater flexibility, as it allows for control over the number of diffusion steps to take and the amount of noise to add. This level of granularity can be advantageous as it gives the flexibility to choose coarse to fine interpolation. In the limiting case of 0 diffusion steps, the interpolation mixes source images in VAE's latent space. On the other hand, as we increase the number of steps, the model will generate more novel samples. This has been demonstrated in the original DDPM paper [(6)](https://sciwheel.com/work/citation?ids=16133297&pre=&suf=&sa=0), where the authors varied the number of diffusion steps prior to interpolation using CelebA data. However, for users with less computational resources, the second method provides a more straightforward and computationally efficient approach. In our assessment, both approaches exhibited comparable performance in capturing correlation shifts (Fig. 3c) throughout the interpolation process.

We have now included in the text the type of interpolation we used, specifically noting that we employed the first approach described in the Methods section for our paper. Additionally, we have provided further clarification and guidance on the selection between these two methods based on their respective advantages and computational considerations.

6. The authors' demonstration of the experimental efficacy of scVAEDer in perturbation prediction requires further scrutiny. scGen (Lotfollahi et al., 2019), claimed to be SOTA, is already a relatively early method, and subsequent methods such as scPreGAN (Wei et al., Bioinformatics 2022), scVIDR(Kana et al., Patterns 2023), CellOT(Bunne et al., Nature methods 2023), etc., have been ignored.

In our work, we primarily compared our method to existing approaches published before April 2023, the time we submitted our manuscript. We've incorporated your suggestion by including additional comparisons with scPreGAN (Figure 4) and the VEGA method. Notably, our results show that scVAEDer surpasses all these methods in terms of accuracy when predicting responses to perturbations. These additions strengthen the validation of scVAEDer's ability to predict such responses. We opted to exclude VEGA from the main figure as its primary advantage lies in interpretability due to the use of a linear activation function, which comes at the cost of significantly lower predictive power compared to the other selected methods.

Also, to further strengthen the validation of our model, we tested its performance on a new perturbation dataset of the intestinal epithelial cells after Salmonella infections and compared it with scGen and scPreGAN (Supplementary Figure 3). This new analysis reinforces scVAEDer's higher accuracy in predicting perturbation responses on this additional data as well.

7. According to the scGen tutorial, it is observed that scGen achieves determination coefficient of mean genes expression in various cell types in the PBMC dataset, mostly above 0.9, even reaching 0.95. However, the results reproduced by the author in Figure 4b appear significantly lower. Did the authors calculate the regression coefficients in Figure 4b using all genes or only a subset of differentially expressed genes (DEGs)?

For comparison between different methods, we did select a subset of genes based on differentially expressed genes (DEGs) analysis, focusing on the top 2000 most significantly altered genes among the compared cell types. scGen paper shows mean gene expression in PBMC data reaching around 0.9, while Figure 4b shows an average similarity of around 0.8. It is important to note that scGen's performance can be sensitive to various factors, including:

1. Gene selection: As you mentioned, the performance and error rate of scGen can indeed be impacted by the set of genes chosen for comparison, as shown in the previous studies (Fig 7. b,c and Supplementary Figure 2) [(7)](https://sciwheel.com/work/citation?ids=10291238&pre=&suf=&sa=0).
2. Hyperparameter tuning: As shown also by the other published studies scGen's performance is sensitive to various hyperparameters, such as the number of layers, activation functions, and distance metric used for comparison [(8)](https://sciwheel.com/work/citation?ids=15815252&pre=&suf=&sa=0). While we have strived to use common and well-established settings, the specific hyperparameter choices in the scGen paper slightly differed from ours to reduce computational complexity (however, we chose the same architecture for both scGen and scVAEDer to have a fair comparison), contributing to the observed difference in performance.

Our findings regarding scGen's performance are consistent with those of other studies that have compared it with similar methods (Bunne et al., 2023). Additionally, we believe a more accurate method for comparing scGen with ours is examining the distribution predictions of different genes, as illustrated in violin plots of Figure 4 and Supplementary Figure 3. As can be seen, scVAEDer consistently outperforms scGen across various genes in terms of predictive accuracy. To elaborate on the performed comparison, we have incorporated further information into the Methods section to provide a clearer explanation.

8. As Figures 4c and 4d do not annotate additional information such as DEGs like scGen, Figure 4b actually encompasses the information present in Figures 4c and 4d. I would recommend the authors utilize low-dimensional visualization to present more details of more cell types.

We have taken your feedback into consideration and have made the necessary adjustments. The scatter plots previously presented in Figures 4c and 4d have been removed. Additionally, we have reorganized Figure 4 and Supplementary Figure 3 to improve clarity and now include the distribution of more key relevant genes known to be important in Lupus disease.

9. It is not clear how the genes were selected for Figure 4e, and the origin of the gene data concerning which cell type is not specified. Additionally, the crucial ISG15 gene, pivotal in perturbation, has not been subjected to comparison.

We have updated Figure 4 and Supplementary Figure 3 to include the crucial ISG gene family, particularly ISG15 and ISG20, aligning with scGen and other relevant literature. Additionally, we conducted an extensive search of published studies and identified genes important in IFN-β perturbation in PBMCs of lupus patients and cited them in our manuscript. We report the model's performance for these genes in the revised analysis. Furthermore, to address your concern regarding the cell type, we have clearly specified it in both the text and the figure legend.

**Minor points:**

1. Typos and tenses should be double checked, such as "we train(ed) new models and showed the stimulation" in Line 23 of Page 10 and "Then the reverse process map(s)" in Lines 17-18 of Page 17.

Thank you for bringing that to our attention. We have thoroughly reviewed the manuscript, and we have made the necessary corrections to address the typos and mistakes.

**Reviewer #2:** In this manuscript, the authors proposed a novel framework, scVAEDer, to learn a representation which can capture both global semantics and local variations. They overcome the limitations of current generative models (VAES, GANs) by employing a Denoising Diffusion Models (DDMs) and combine the strengths of VAEs and DDMs. Then it can be used for multiple downstream tasks, such as data generation, cell differentiation, prediction of gene expression and detection of master regulators. They tested the model on different datasets and demonstrated that scVAEDer outperformed most competing methods across most datasets. However, the results from the conducted experiments are not sufficient and compelling, which fail to demonstrate the significance of scVAEDer and its advantages over the existing methods. Detailed problems are given as follows.

**Major**

1. The authors only provided a case analysis 'interpolate between monocytes to Hematopoietic Stem/Progenitor Cells (HSPC)' in the part of scVAEDer models the transition between cells.

I suggest that the authors provide references or a detailed explanation as to why this specific cell transition (from monocytes to Hematopoietic Stem/Progenitor Cells) was chosen as the study case. If the aim of the article is to demonstrate the general applicability of the scVAEDer method, including a broader range of cell transitions might make the argument more convincing.

We appreciate the reviewer's insightful comment. Our initial focus on the transformation from monocytes to Hematopoietic Stem/Progenitor Cells (HSPCs) stemmed from the acknowledged significance and complex nature of this transition in reprogramming research [(9)](https://sciwheel.com/work/citation?ids=16201413&pre=&suf=&sa=0). In response to your valuable feedback, we have incorporated a detailed explanation and relevant references within the manuscript. This explanation highlights the crucial role of monocytes as a central component of the host's immune system and underscores the inherent complexities associated with generating monocyte-derived induced pluripotent stem cells (iPSCs).

2. The authors only provided limited evidence to show the advantages of the proposed model over the state-of-the-art methods.

In all downstream analyses, apart from the section 'scVAEDer predicts perturbation responses using feature manipulation', there is no comparison with state-of-the-art (SOTA) methods in the other sections. Moreover, this analysis only chose one method, scGen, and a single dataset (PBMCs) for comparison, does not provide substantial evidence to assert that scVAEDer is superior to other methods. Additionally, are there limitations in the types of single-cell transcriptomics data it can handle? If not, why is each experiment conducted using only one dataset? Please give more description.

We've taken into account your concerns and made the following adjustments: Firstly, in response to your suggestion, we have broadened our comparative analysis by including two additional methods, namely scPREGAN and VEGA, both of which were published prior to April 2023, the submission date of our manuscript. Notably, our results show that scVAEDer surpasses all these methods in terms of accuracy when predicting responses to perturbations. These additions strengthen the validation of scVAEDer's ability to predict such responses. We opted to exclude VEGA from the main figure as its primary advantage lies in interpretability due to the use of a linear activation function, which comes at the cost of significantly lower predictive power compared to the other selected methods. These additions are now reflected in the revised manuscript.

Additionally, to further strengthen the validation of our model, we tested the performance of our model on a new perturbation dataset (human peripheral blood mononuclear cells stimulated with interferon) and compared it with scGen and scPreGAN (Supplementary Figure 3). This new analysis reinforces scVAEDer's superior accuracy in predicting perturbation responses on this additional dataset as well.

Secondly, it's important to note that VAE is a common method in the field, for tasks such as interpolation, data generation and perturbation prediction [(10–13)](https://sciwheel.com/work/citation?ids=14800044,7244120,14466219,14422604&pre=&pre=&pre=&pre=&suf=&suf=&suf=&suf=&sa=0,0,0,0). As stated in the introduction of our paper and shown by previous works in different areas of machine learning, VAE models are subject to several limitations. Therefore we provide a comprehensive evaluation of our model for different tasks. We first compared the performance of scVAEDer against VAE in terms of data generation accuracy and the preservation of data structure. Our results show that scVAEDer significantly outperforms VAEs in data generation quality and accuracy (Figure 2e), as well as the preservation of data structure (Figure 2c,d).

Regarding interpolation tasks, prior studies have highlighted the significant limitations in interpolating between different encodings within VAE latent embeddings [(2,5)](https://sciwheel.com/work/citation?ids=14630581,14630616&pre=&pre=&suf=&suf=&sa=0,0). For instance, the phenomenon known as the "prior hole" problem can lead to artifacts and unrealistic representations when transitioning between states. To address this issue, scVAEDer performs interpolations within the DDM's latent space rather than the VAE's. This approach avoids potential artifacts that arise from traversing "empty" regions in the VAE's space during transitions between different encodings consistent with other studies (figure 3b of our paper).

While there is no definitive gold standard list of master regulators for different biological processes such as development and differentiation, we have employed established practices to validate our model's performance in detecting master regulators. We have tested the model's performance by comparing it with previously reported key genes identified through multiple experiments and conducted gene set enrichment analysis to demonstrate the significance of the detected genes in these specific pathways (Figure 5 and Supplementary Table 1). These approaches align with established practices in the field [(12,14–16)](https://sciwheel.com/work/citation?ids=14346796,12387806,14466219,16133457&pre=&pre=&pre=&pre=&suf=&suf=&suf=&suf=&sa=0,0,0,0).

We have made modifications in the text and specified that we have used four different datasets with various sizes and model organisms to assess the ability of scVAEDer in generation, interpolation, prediction and detection of master regulators. We showed the power of scVAEDer in data generation as it can preserve the structure of the data and generate high-quality samples from just a Gaussian noise (Figures 2, 3b&c, 4, 5a&c&d , Supplementary Figure 2)

3. The authors only provided an analysis of the data generation capabilities of VAEs and DDMs, but the absence of any ablation studies in the paper.

This manuscript lack of the ability to validate the effectiveness of these two components within the model. For instance, it remains unclear how the results would vary if only the DDM model were used, or if a module with similar functionality were to replace the VAE. This lack of empirical evidence makes it challenging to conclusively demonstrate that the integration of both VAE and DDM is essential for the model's performance.

While DDMs like DDPM [(17)](https://sciwheel.com/work/citation?ids=12653859&pre=&suf=&sa=0) and DDIM [(18)](https://sciwheel.com/work/citation?ids=14557049&pre=&suf=&sa=0) are powerful for generating high-quality data, their application to high-dimensional datasets like single-cell RNA-seq presents a significant computational bottleneck. Training and evaluating such models involve repeated, computationally expensive operations in the high-dimensional space of the data. Published research indicates that even for low-dimensional image generation tasks, training diffusion models can require astronomical resources, consuming tens of GPU days (e.g., 150-1000 V100 days) and data generation exceeding 5 days on A100 GPUs [(1,3)](https://sciwheel.com/work/citation?ids=13075114,14630596&pre=&pre=&suf=&suf=&sa=0,0). Unfortunately, despite our efforts to gain access to A100 machines in Microsoft's cloud services and even getting access to their Tesla M60 machines, training such models requires massive computational resources that are not available to the academic community or not feasible at all. Also, DDM models alone lack a low-dimensional latent representation, limiting their practical applicability for downstream applications of high-dimensional data.

Regarding changing the VAE to other representational learning methods, It's worth noting that this remains an open question in the field of diffusion models. Previous studies have used different methods for representation learning such as AE, VAEs, beta-VAE, and infoVAE [(3,4,19,20)](https://sciwheel.com/work/citation?ids=16194128,14630596,13328793,16194134&pre=&pre=&pre=&pre=&suf=&suf=&suf=&suf=&sa=0,0,0,0). Therefore, we are actively working to address this question in our future study. We plan to explore how substituting the VAE with different representation learning techniques can potentially enhance the accuracy and efficiency of scVAEDer. Additionally, investigating causal representation learning may further enhance the performance of scVAEDer by capturing both the data's structure and the underlying relationships between variables. To partially address your question we conducted a preliminary study where we replaced the VAE with a Beta-VAE in Zebrafish hematopoiesis. We observed that the data generation process resulted in a total variation distance (TVD) of approximately 0.52, which, although better than the VAE (blue column in Figure 2e), did not match the performance of scVAEDer (Orange column in Figure 2e). It is noteworthy that the Beta-VAE has an additional feature of disentanglement between latent dimensions.

We have now revised the text to provide additional insights into the computational complexity of deep diffusion models. Furthermore, we have expanded the discussion to include the potential and significance of other representation learning methods.

4. In the "Model Architecture and Training Process" section, the authors fail to provide sufficient details about the model, raising concerns about the replicability of the experiment.

Firstly, there is a lack of specific details regarding the hyperparameter search, such as the search space, algorithms used, and evaluation criteria, all of which are crucial for assessing the model's performance and reproducibility. Secondly, while the trade-off between performance and computational resources is mentioned, the authors do not elaborate on how this balance was achieved, nor do they discuss how it impacts the final performance of the model.

To address concerns about replicability and hyperparameter optimization, the manuscript has been revised to include a detailed description of the hyperparameter search process (Methods section, Supplementary Tables 2 and 3). This description includes the search space, algorithms used, and evaluation criteria used.

Additionally, a new section has been incorporated into the manuscript to show the trade-off between model performance, available computational resources and overfitting issue. Specifically, we examine how varying the number of diffusion steps and the architecture of DDMs can affect both data quality and processing time, which provides insights into optimizing scVAEDer's performance within the constraints of computational resources (Methods). Moreover, We have included additional insights on choosing the most suitable interpolation approach, considering users' objectives and available computational resources.

5. The article does not include extensive validation of the scVAEDer model using benchmark datasets or real biological data, raising concerns about its generalizability and reliability.

We appreciate all your valuable feedback. Following your suggestions, we have integrated a new biological dataset into our study and conducted additional analysis and comparisons. We also modified the text description for clarification. These adjustments were made to address concerns regarding the robustness and generalizability of scVAEDer. We hope that these changes have strengthened the reliability of our findings.

**Minor**

1. Regarding Fig.1, the unclear workflow presented can easily lead to confusion for readers. For instance, it is not apparent whether the training proceeds from top to bottom or from left to right. Clarifications are needed on where the data input is located, and which variable units interact between the VAE and DDM.

We apologize for the confusion regarding Figure 1. We have updated both the figure and the accompanying text to ensure clarity and readability and to address the issues you raised.

2. There is a discrepancy between the textual description and the figure representation. The text mentions that is used to train a diffusion model, yet Fig.1 illustrates X. This inconsistency

needs to be addressed to ensure coherence and understanding.

We've corrected the discrepancy between the text and the figure.

3. In the "Data Availability" section, there is no mention of the PBMC dataset used in the part of the study where scVAEDer predicts perturbation responses through feature manipulation.

We've addressed this by including the PBMC data along with the newly added dataset. Additionally, we've ensured proper citations for all datasets used in the study.

4. It is recommended that the authors present statistical results from multiple experiments to validate the robustness of the model.

Considering the computational complexity of the model, we opted for additional validation methods to ensure its robustness. Our approach involved assessing various aspects: first, we examined the quality of simulated samples by scVAEDer, as a generative model. Next, we meticulously evaluated the prediction accuracy of holdout samples during perturbation using different datasets. Lastly, and perhaps most crucially, we conducted an extensive analysis to gauge the model's performance in capturing the important and relevant biological signals. This involved an extensive search across key genes and pathways. Through this comprehensive approach using multiple biological datasets, we aimed to solidify the validity and reliability of scVAEDer.

5. Fig.5(g) is not cited in the main text, and it lacks a detailed biological interpretation and relevance of its results. Providing examples of novel biological discoveries or confirming known phenomena would significantly strengthen the manuscript

Thank you for bringing that to our attention. We've addressed both points by adding a proper citation in the text and adding a detailed explanation of the biological significance of the identified genes by our method (Result section, Supplementary Table 1).

**References**

[1.	Rombach R, Blattmann A, Lorenz D, Esser P, Ommer B. High-Resolution Image Synthesis with Latent Diffusion Models. arXiv. 2021;](https://sciwheel.com/work/bibliography/13075114)

[2.	Zeng X, Vahdat A, Williams F, Gojcic Z, Litany O, Fidler S, et al. LION: Latent Point Diffusion Models for 3D Shape Generation. arXiv. 2022;](https://sciwheel.com/work/bibliography/14630581)

[3.	Pandey K, Mukherjee A, Rai P, Kumar A. DiffuseVAE: Efficient, Controllable and High-Fidelity Generation from Low-Dimensional Latents. arXiv. 2022;](https://sciwheel.com/work/bibliography/14630596)

[4.	Preechakul K, Chatthee N, Wizadwongsa S, Suwajanakorn S. Diffusion Autoencoders: Toward a Meaningful and Decodable Representation. arXiv. 2021;](https://sciwheel.com/work/bibliography/13328793)

[5.	Kreis K, Dockhorn T, Li Z, Zhong E. Latent Space Diffusion Models of Cryo-EM Structures. arXiv. 2022;](https://sciwheel.com/work/bibliography/14630616)

[6.	Ho J, Jain A, Abbeel P. Denoising diffusion probabilistic models. Advances in neural information processing systems. 2020;33:6840–51.](https://sciwheel.com/work/bibliography/16133297)

[7.	Lotfollahi M, Naghipourfar M, Theis FJ, Wolf FA. Conditional out-of-distribution generation for unpaired data using transfer VAE. Bioinformatics. 2020 Dec 30;36(Supplement_2):i610–7.](https://sciwheel.com/work/bibliography/10291238)

[8.	Ji Y, Green T, Peidli S, Bahrami M, Liu M, Zappia L, et al. Optimal distance metrics for single-cell RNA-seq populations. BioRxiv. 2023 Dec 27;](https://sciwheel.com/work/bibliography/15815252)

[9.	Isogai S, Yamamoto N, Hiramatsu N, Goto Y, Hayashi M, Kondo M, et al. Preparation of induced pluripotent stem cells using human peripheral blood monocytes. Cell Reprogram. 2018 Dec;20(6):347–55.](https://sciwheel.com/work/bibliography/16201413)

[10.	Lotfollahi M, Klimovskaia Susmelj A, De Donno C, Hetzel L, Ji Y, Ibarra IL, et al. Predicting cellular responses to complex perturbations in high-throughput screens. Mol Syst Biol. 2023 Jun 12;19(6):e11517.](https://sciwheel.com/work/bibliography/14800044)

[11.	Lotfollahi M, Wolf FA, Theis FJ. scGen predicts single-cell perturbation responses. Nat Methods. 2019 Aug;16(8):715–21.](https://sciwheel.com/work/bibliography/7244120)

[12.	Sadria M, Layton A, Goyal S, Bader G. Fatecode: Cell fate regulator prediction using classification autoencoder perturbation. BioRxiv. 2022 Dec 19;](https://sciwheel.com/work/bibliography/14466219)

[13.	Choi Y, Li R, Quon G. siVAE: interpretable deep generative models for single-cell transcriptomes. Genome Biol. 2023 Feb 20;24(1):29.](https://sciwheel.com/work/bibliography/14422604)

[14.	Kamimoto K, Stringa B, Hoffmann CM, Jindal K, Solnica-Krezel L, Morris SA. Dissecting cell identity via network inference and in silico gene perturbation. Nature. 2023 Feb 8;614(7949):742–51.](https://sciwheel.com/work/bibliography/14346796)

[15.	Osorio D, Zhong Y, Li G, Xu Q, Yang Y, Tian Y, et al. scTenifoldKnk: An efficient virtual knockout tool for gene function predictions via single-cell gene regulatory network perturbation. Patterns (N Y). 2022 Mar 11;3(3):100434.](https://sciwheel.com/work/bibliography/12387806)

[16.	Yang Y, Li G, Zhong Y, Xu Q, Chen B-J, Lin Y-T, et al. Gene knockout inference with variational graph autoencoder learning single-cell gene regulatory networks. Nucleic Acids Res. 2023 Jul 21;51(13):6578–92.](https://sciwheel.com/work/bibliography/16133457)

[17.	Ho J, Jain A, Abbeel P. Denoising Diffusion Probabilistic Models. arXiv. 2020;](https://sciwheel.com/work/bibliography/12653859)

[18.	Song J, Meng C, Ermon S. Denoising Diffusion Implicit Models. arXiv. 2020;](https://sciwheel.com/work/bibliography/14557049)

[19.	Batzolis G, Stanczuk J, Schönlieb CB. Variational Diffusion Auto-encoder: Latent Space Extraction from Pre-trained Diffusion Models. arXiv preprint arXiv:230412141. 2023;](https://sciwheel.com/work/bibliography/16194128)

[20.	Wang Y, Schiff Y, Gokaslan A, Pan W, Wang F, De Sa C, et al. Infodiffusion: Representation learning using information maximizing diffusion models. International Conference on Machine Learning. 2023;36336.](https://sciwheel.com/work/bibliography/16194134)

**2^nd^ round**

**Reviewer 2**

Many issues have been addressed and the following problems remain.

Major 2: The decision to exclude VEGA from the main figure is rational given its focus on interpretability rather than predictive power. However, it would be beneficial to include a brief discussion or a supplementary figure to illustrate VEGA's performance for readers interested in the trade-offs between interpretability and predictive accuracy. Alternatively, a justification for why this method was included as a competing method should be provided if it is not suited for predictive purposes. Additionally, I did not find any mention of the VEGA method anywhere in the manuscript.

About Validation of Master Regulators Detection: The methods used to validate the detection of master regulators seem appropriate. It could enhance the manuscript if the authors discussed any discrepancies between their findings and previous reports, if applicable, and how these differences contribute to the field.

Major 3: The authors have adequately discussed and partially resolved the issues I raised. Indeed, as they mentioned, the DDM module's proficiency in generating high-quality and high-resolution images or other complex data types may lead to computational bottlenecks. However, the paper still lacks experimental results to quantify the effectiveness of the DDM. Specifically, it does not clarify why this study must use DDM instead of other generative models. Could we consider the following approaches to address this? 1. Replace the DDM module and compare the performance of different models. 2. Adjust key parameters of the DDM to observe how these changes affect the outcomes. 3. Alter the characteristics of the input data (by adding noise or changing the data distribution) to test the model's sensitivity to these changes. These methods could help understand the robustness of the model and the contribution of the DDM module.

**Authors’ response**

We thank the reviewer for the insightful comments. Please find our responses and the corresponding changes below.

Reviewer #2: Many issues have been addressed and the following problems remain.

Major 2: The decision to exclude VEGA from the main figure is rational given its focus on interpretability rather than predictive power. However, it would be beneficial to include a brief discussion or a supplementary figure to illustrate VEGA's performance for readers interested in the trade-offs between interpretability and predictive accuracy. Alternatively, a justification for why this method was included as a competing method should be provided if it is not suited for predictive purposes. Additionally, I did not find any mention of the VEGA method anywhere in the manuscript.

Thank you for your valuable suggestion. We have now cited the VEGA method in the Methods section, clarifying its performance and stating that it emphasizes interpretability over predictive power. In selecting methods for inclusion when writing our manuscript, we aimed to represent state-of-the-art approaches in single-cell RNA-seq gene expression prediction following perturbation. While VEGA claims to achieve both interpretability and accuracy, similar to scGen and scPreGAN, our analysis found that VEGA’s predictive performance was comparatively lower. Nonetheless, we believe that including VEGA will be beneficial for readers interested in exploring interpretability-focused methods alongside more predictive ones for perturbation prediction tasks.

About Validation of Master Regulators Detection: The methods used to validate the detection of master regulators seem appropriate. It could enhance the manuscript if the authors discussed any discrepancies between their findings and previous reports, if applicable, and how these differences contribute to the field.

We agree that understanding whether the detected genes are specific to this particular biological process—lineage reprogramming of mouse embryonic fibroblasts (MEFs) to induced endoderm progenitors (iEPs)—or if they are pivotal across other related processes would be valuable. We also believe that additional studies on diverse datasets would be beneficial to clarify the relationship between genes with high expression and key master regulators in various biological contexts. We have incorporated these points into the discussion to highlight the need for broader validation and exploration of master regulators across multiple processes.

Major 3: The authors have adequately discussed and partially resolved the issues I raised. Indeed, as they mentioned, the DDM module's proficiency in generating high-quality and high-resolution images or other complex data types may lead to computational bottlenecks. However, the paper still lacks experimental results to quantify the effectiveness of the DDM. Specifically, it does not clarify why this study must use DDM instead of other generative models. Could we consider the following approaches to address this? 1. Replace the DDM module and compare the performance of different models. 2. Adjust key parameters of the DDM to observe how these changes affect the outcomes. 3. Alter the characteristics of the input data (by adding noise or changing the data distribution) to test the model's sensitivity to these changes. These methods could help understand the robustness of the model and the contribution of the DDM module.

Thank you for your thoughtful feedback. The power and novelty of our method largely stem from the DDM module, as without it, the model would closely resemble scGen, relying solely on an autoencoder structure. To emphasize the importance of the DDM, we conducted several perturbation prediction tasks using different datasets, showing that our model, scVAEDer, significantly outperforms autoencoder-based methods (scGen) as well as GAN-based methods (scPreGAN). Also, we performed a hyperparameter search on key parameters, including noise scheduling functions and the number of diffusion steps (see Supplementary Table 3), to examine how variations in these settings affect performance. We have expanded the manuscript to emphasize these experimental adjustments and to highlight the influence of DDM and related parameters on the power and predictive capabilities of scVAEDer.

**3^rd^ round**

**Reviewer 2**

The revisions and responses from R2 have successfully addressed the issues I previously raised.
